# Small molecule targeting long noncoding RNA GAS5 administered intranasally improves neuronal insulin signaling and decreases neuroinflammation in an aged mouse model

**DOI:** 10.1038/s41598-022-27126-6

**Published:** 2023-01-06

**Authors:** Rekha S. Patel, Ashley Lui, Charles Hudson, Lauren Moss, Robert P. Sparks, Shannon E. Hill, Yan Shi, Jianfeng Cai, Laura J. Blair, Paula C. Bickford, Niketa A. Patel

**Affiliations:** 1 James A. Haley Veterans Hospital, Research Service, 13000 Bruce B. Downs Blvd., Tampa, FL 33612 USA; 2 Department of Molecular Medicine, University of South Florida, Tampa, FL 33612 USA; 3 Department of Neurosurgery and Brain Repair, University of South Florida, Tampa, FL 33612 USA; 4 Department of Chemistry, University of South Florida, Tampa, FL 33612 USA; 5 USF Health Byrd Institute, University of South Florida, Tampa, FL 33612 USA; 6 Present Address: UMass Chan Medical School, Worcester, MA 01655 USA

**Keywords:** Non-coding RNAs, Geriatrics, Molecular biology, Molecular medicine

## Abstract

Shifts in normal aging set stage for neurodegeneration and dementia affecting 1 in 10 adults. The study demonstrates that lncRNA GAS5 is decreased in aged and Alzheimer’s disease brain. The role and targets of lncRNA GAS5 in the aging brain were elucidated using a GAS5-targeting small molecule NPC86, a frontier in lncRNA-targeting therapeutic. Robust techniques such as molecular dynamics simulation of NPC86 binding to GAS5, in vitro functional assays demonstrating that GAS5 regulates insulin signaling, neuronal survival, phosphorylation of tau, and neuroinflammation via toll-like receptors support the role of GAS5 in maintaining healthy neurons. The study demonstrates the safety and efficacy of intranasal NPC86 treatment in aged mice to improve cellular functions with transcriptomic analysis in response to NPC86. In summary, the study demonstrates that GAS5 contributes to pathways associated with neurodegeneration and NPC86 has tremendous therapeutic potential to prevent the advent of neurodegenerative diseases and dementias.

## Introduction

With advances in medicine and technology, humans are living longer. It is estimated that 1.4 billion people are over the age of 60 worldwide. According to the US Census, older adults are projected to be 23.4% of the population by 2060. Healthy aging is associated with slight decreases in overall body capabilities including mild decreases in cognition. However, shifts in normal aging set the stage for neurodegeneration and cognitive disorders including dementia. Epidemiological and clinical studies have overwhelmingly confirmed that defective insulin signaling in the brain plays a central role in the early stages of dementia, a primary feature of sporadic Alzheimer’s disease (AD) and AD- Related Dementias (ADRD) pathology^1–9^.

It is projected that by 2050, 106.23 million adults will be living with AD worldwide. In the USA, 1 in 10 adults over the age of 65 have AD (Source: Alzheimer’s Association Report 2020). Dementia and particularly Alzheimer’s disease affects almost 25% of US veterans over the age of 65 years and this number doubles in the group above 75 years. Additionally, sedentary lifestyles during post-service years dramatically increase the prevalence of type 2 diabetes (T2D) and insulin resistance which increase the risk for impaired cognitive function (affecting verbal and nonverbal memory) and dementia by 47%^10,11^. Of particular importance are the Vietnam War veterans exposed to Agent Orange who have a 79% increased risk of dementia with T2D as the predominant risk factor compared to other veterans^12–16^. The shift from normal aging to neurodegeneration is a long process with slow progression of individual symptoms such as cognitive decline or memory losses. Efforts to treat the pathology of AD at late stages have not been effective in clinic^17–19^. Hence, there is an urgent need to target the modifiable, early risk factors during aging and thereby prevent the onset of neurodegeneration.

Long noncoding RNA (lncRNA) are the largest subset of noncoding RNA found in the human genome. Recent discoveries have highlighted its multi-faceted, central role as regulators of gene expression and cellular function. It is now established that dysregulation of lncRNA expression plays an integral part in manifestation of human diseases^20–23^. The lncRNA growth-arrest specific transcript 5 (GAS5) is shown to regulate cell growth, proliferation and survival^24,25^. We previously demonstrated that GAS5 levels are decreased consistently in humans with type 2 diabetes, an insulin resistant state^26^. Our extensive prior research demonstrated that GAS5 regulates the expression of insulin receptor and insulin signaling pathway in diabetic adipocytes^27^. Other studies along with research from our lab^28^ has demonstrated the role of GAS5 in mediating an inflammatory response. Since insulin signaling and inflammation play a critical role in neuronal metabolic pathways and cognition, we evaluated the expression of GAS5 in the aging brain. Our results demonstrate that GAS5 expression is decreased in human brain tissue of older and AD subjects. Additionally, we demonstrate that neuronal GAS5 is decreased in brain of aged compared to young mice as well as in diet-induced obese (insulin resistant) mice compared to age-matched normal mice. We previously reported the development of lncRNA GAS5-targeting small molecule called NPC86, a frontier in the field of RNA-targeted drug discovery^27^. The role of GAS5 in the aging brain is lesser known and hence we sought to systematically evaluate GAS5 using a multi-disciplinary approach integrating molecular dynamics, transcriptomics and in vivo evaluation of effects of NPC86 treatment on GAS5 levels. Hence, we administered NPC86 intranasally to aged mice and elucidate its uptake in the brain. NPC86 is non-toxic and increases GAS5 levels in the brain of aged mice. Further, we demonstrate improved neuronal insulin signaling and modulation of neuroinflammation in vivo. To understand the transcriptomic changes, we performed RNAseq on samples from the hippocampus of treated mice. Results identify genes whose expression were altered in aged mice in a NPC86-dependent manner and novel targets of GAS5 were identified.

## Results

### GAS5 levels in brain

**Figure 1:**
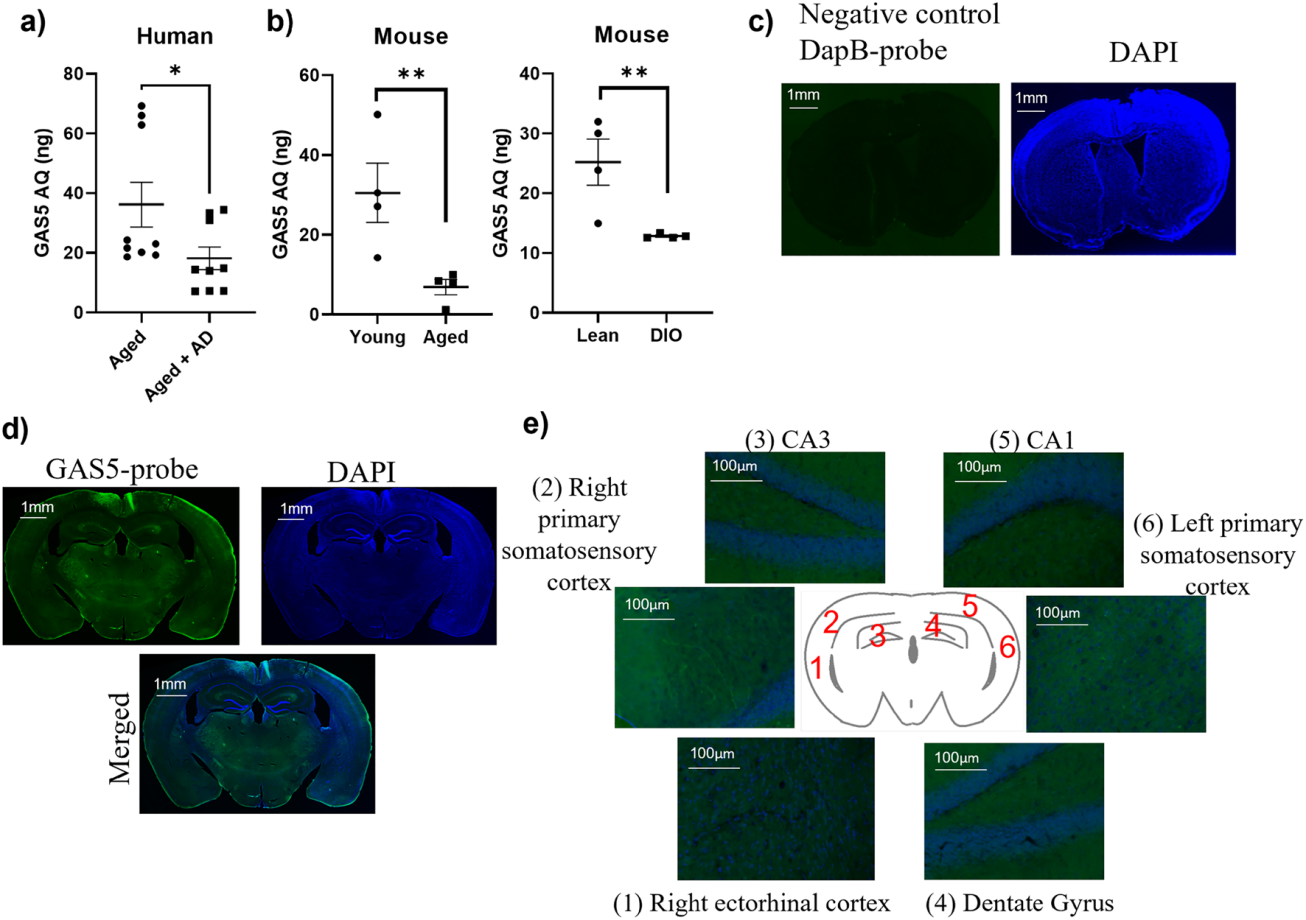
GAS5 levels in brain (**a**) QPCR was performed on RNA isolated from human temporal lobe from aged and aged-matched patients with Alzheimer’s Disease (no cancer, aged 70–85 yr, M/F, n = 9 each). A standard curve was generated for GAS5 expression levels and absolute quantification (AQ; ng) was calculated and normalized to β-actin expression. Statistical analysis was performed by two-tailed Student’s t-test, *p < 0.05. (**b**) Hippocampal tissue was harvested from C57BL6 mice 6-month-old mice (Young) or 20 month old mice (Aged), or littermates fed with control chow (Lean) or high fat diet to generate diet induced obese mice (DIO). RNA was isolated and qPCR was performed for GAS5 levels. A standard curve was generated for GAS5 expression levels and absolute quantification (AQ, ng) was performed normalized to β-actin expression (n = 4). Statistical analysis was performed by two-tailed Student’s t-test, **p < 0.01. (**c**) All sections were treated with Sudan black B (SBB) dye to eliminate autofluorescence and nuclear counterstained with DAPI. DapB was used as negative control probe in RNAscope. (**d**) Representative image of GAS5 in situ hybridization (GAS5-ISH) in whole mouse brain section. Scale bar represents 1 mm e) Representative 20 × images of GAS5-ISH insets of the (1) ectorhinal cortex, (2) and (6) primary somatosensory cortex, (3) CA3, (4) dentate gyrus, and (5) CA1 are shown.; inset scale bar represents 100 µm.

To evaluate the levels of GAS5 in the brain, RNA from human medial temporal lobe from Alzheimer’s disease (AD) patients or age matched normal (no cancers, 70–85 years, male and female, n = 9; from Byrd Alzheimer’s Institute) were obtained and analyzed using real-time qPCR. Results (Fig. 1a) show that GAS5 is present in the aging brain but levels are reduced in human AD samples.

Next, we evaluated GAS5 levels in hippocampus of young (6 months) and aged (20 months) C57BL/6J mice. Results (Fig. 1b) from real time qPCR demonstrated that aged mice had lower GAS5 levels compared to young mice. Since our prior research had showed that GAS5 was lower in diabetes^26^, we determined the GAS5 levels in the hippocampus of diet-induced obese (DIO) mice (age 6 months; diabetic). Interestingly, the DIO mice had significantly lower GAS5 levels in brain compared to normal, lean non-diabetic mice.

Previous studies using single cell RNA-FISH have shown that GAS5 shuttles between the cytosol and nucleus^29,30^. Here, we determined the expression across the mouse brain regions using GAS5 in situ hybridization (RNAscope; ACD). Results (Fig. 1c,d) show that GAS5 is expressed throughout the brain and the magnified images (Fig. 1e) demonstrate ectorhinal cortex, primary somatosensory cortex, CA1, CA3 and dentate gyrus express GAS5 abundantly.

### Depletion of GAS5 reduces insulin receptor levels and insulin signaling in neurons

**Figure 2:**
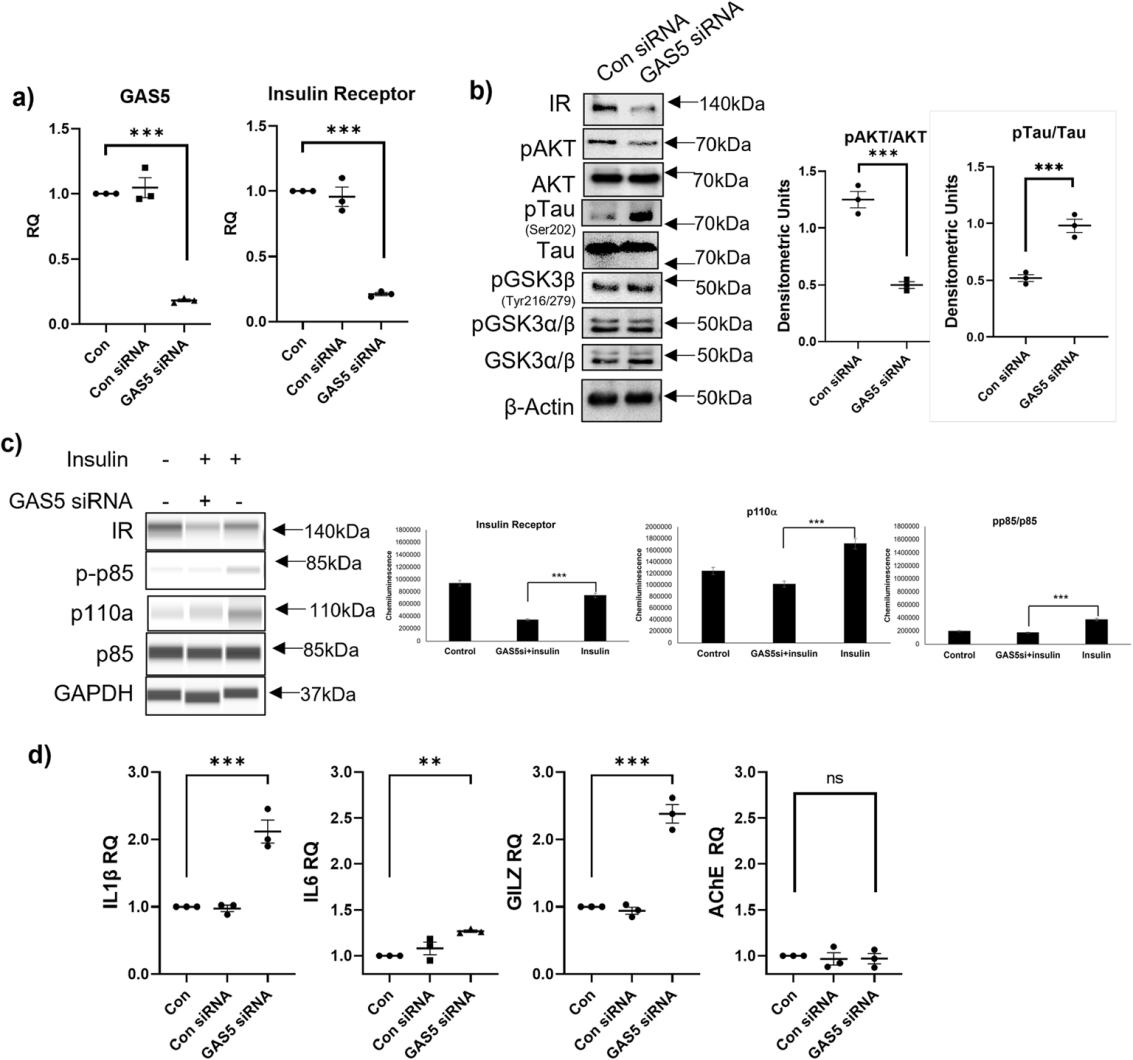
Depletion of GAS5 reduces insulin receptor levels and insulin signaling in neurons. HT22 cells were transfected with GAS5 siRNA or scrambled control siRNA (Con siRNA) for 48 h. (**a**) RNA was isolated, qPCR was performed, normalized to β-actin expression and relative quantification (RQ) was determined for GAS5 and IR levels using control as reference (n = 3). Statistical analysis was performed by one-way ANOVA, ***p < 0.001. (**b**) Cell lysate was harvested, and western blot was performed using antibodies against IR, pAKT, AKT, pTau, Tau, pGSK3β, pGSK3α/β, GSK3α/β, and β-actin. Graph shows relative densitometric analysis of individual bands as indicated with phosphorylated protein normalized to total protein (n = 3). Statistical analysis was performed by two-tail Student’s t-test, ***p < 0.001. (**c**) HT22 cells were transfected with GAS5 siRNA for 24 h followed by treatment with 100 nM insulin for 24 h. Whole cell lysates were analyzed using automated WES with antibodies against IR, p-p85, p85, p110α, GAPDH and the virtual blots generated by WES software COMPASS are shown. The graph shows chemiluminescence peaks normalized to GAPDH generated by COMPASS (n = 3). Statistical analysis was performed by two-tail Student’s t-test, ***p < 0.001. (**d**) QPCR was performed, normalized to β-actin expression and relative quantification (RQ) determined for IL1β, IL6, GILZ, and AChE levels using control as reference (n = 3). Statistical analysis was performed by one-way ANOVA,**p < 0.01, ***p < 0.001, and ns not significant.

Our previous research in human adipocytes had indicated that GAS5 regulated the expression of insulin receptor (IR) and insulin signaling pathway^27^. To evaluate whether GAS5 also affected IR levels in the neurons, HT22 cells (mouse hippocampal) were transfected with GAS5 siRNA. Real-time qPCR results (Fig. 2a) showed that depleting GAS5 levels significantly reduced IR levels. Separately, whole cell lysates were collected from GAS5 siRNA transfected cells and results by western blot (Fig. 2b) show decrease in insulin receptor and phosphorylation of Akt, a downstream mediator of insulin signaling.

### GAS5 modulates phosphorylation of tau

Phosphorylation of tau in neurons plays an integral part in the pathology of AD. GAS5 siRNA was transfected into HT22 cells as described above. Whole cell lysates were analyzed for phosphorylation of tau by western blot. Results (Fig. 2b) show that depletion of GAS5 resulted in increase in phosphorylation of tau concurrent with decrease in pAKT. GSK3α and β are implicated in phosphorylation of tau and hence we evaluated their phosphorylation levels. Results show that depletion of GAS5 did not affect the phosphorylation or total levels of GSK3α/β.

### GAS5 is required for insulin signaling

To validate that GAS5 levels affect insulin signaling pathway, GAS5 was depleted using GAS5-siRNA in HT22 cells followed by 100 nM insulin treatment for 24 h. Whole cell lysates were analyzed by automated western blot (WES). Results (Fig. 2c) show robust phosphorylation of phosphatidylinositol 3-kinase (PI3K) regulatory subunits p85 and p110 in response to insulin stimulation. Depletion of GAS5 significantly attenuated response to insulin treatment with decreased phosphorylation of p85 and p110.

### Depletion of GAS5 increases pro-inflammatory genes in neurons

Prior research from our lab^28^ and others^30–32^ have shown that GAS5 modulates inflammatory pathways. Hence, we evaluated the effect of depleting GAS5 using siRNA in HT22 cells on inflammatory pathway genes. Our results (Fig. 2d) demonstrate that depletion of GAS5 resulted in concurrent increase in pro-inflammatory interleukins IL1β and IL6. GAS5 sequesters glucocorticoid receptor (GR)^30^ and hence we evaluated the effect of depleting GAS5 on expression of GR-target gene glucocorticoid inducible leucine-zipper (GILZ). Results (Fig. 2d) demonstrate that GAS5 depletion results in increased expression of Gilz. Depletion of GAS5 had no effect on acetylcholine esterase (AChE) levels thereby demonstrating specificity in the mode of action of GAS5.

### Small molecule NPC86 binds robustly to GAS5 and increases GAS5 and insulin receptor levels in vitro

**Figure 3:**
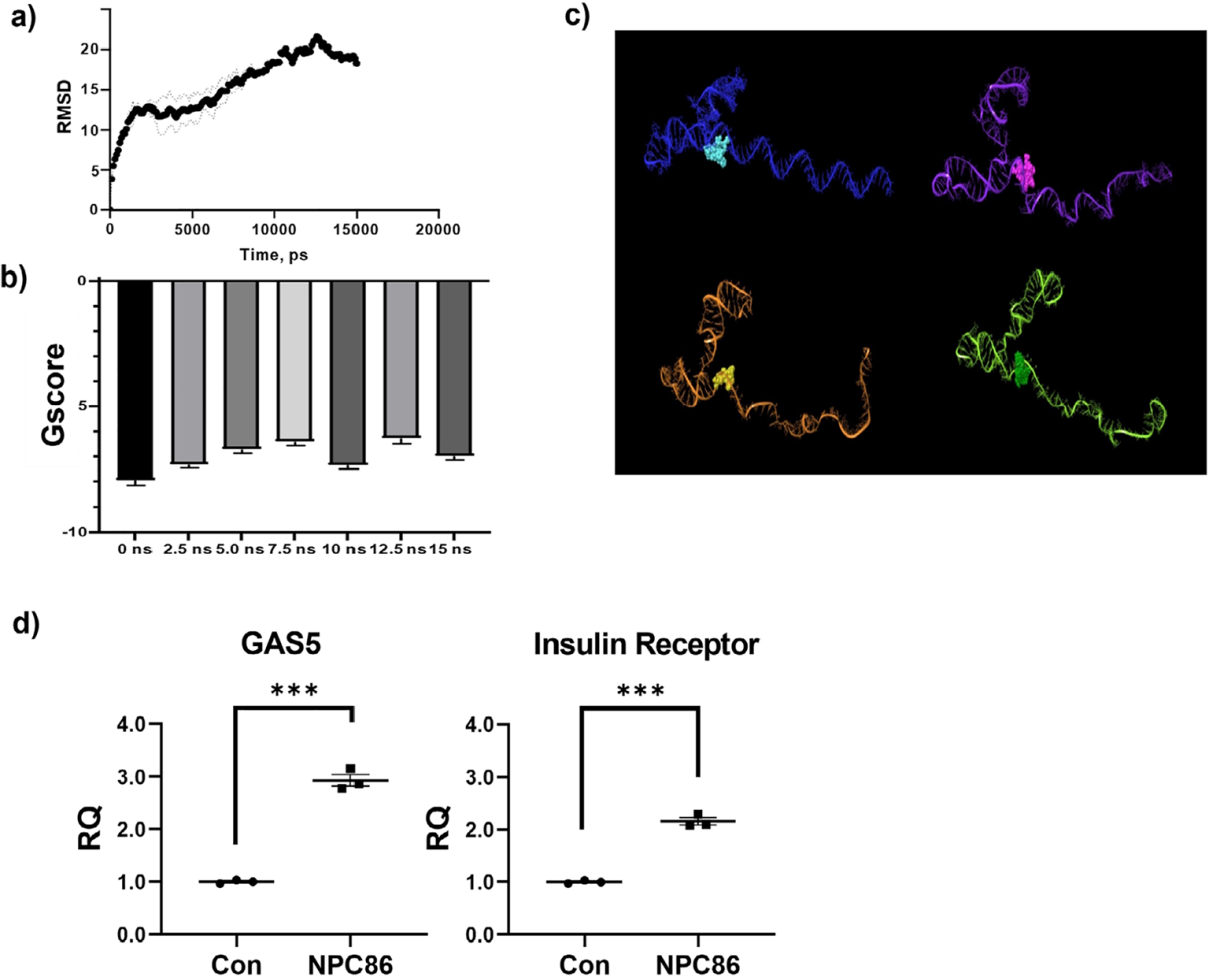
(**a**) RMSD over the course of molecular dynamics equilibration simulations of 10 and 15 ns taken from backbone of RNA structure to the first starting pose show conformation stabilization between 2.5 and 7.5 ns. (**b**) Gscore analysis demonstrates docking of NPC86 to GAS5 transcript using Schrodinger Glide SP showing a lowered predicted affinity during the transition from 0–7.5 ns. (**c**) Slices of the docking simulation exported and visualized in Schrodinger software taken at 0 ns (blue), 5 ns (purple), 10 ns (orange), and 15 ns (green) with NPC86 docked to each slice at the predicted binding region. Two loops of GAS5 near the binding site of NPC86 show flexibility, where NPC86 appears to bind near a hinge region in between these loops. (**d**) RNA was isolated from HT22 cells treated with 20 nM NPC86 and qPCR performed using primers specific to GAS5 and insulin receptor and normalized to β-actin levels. Relative quantification (RQ) was determined using control as reference (n = 3). Statistical analysis was performed by two-tail Student’s t-test, ***p < 0.001.

The above results demonstrate that lncRNA GAS5 is a viable target in neurons to modulate metabolic pathways and neuroinflammation. We previously developed a GAS5 targeting small molecule (NPC86) that increases GAS5 levels in adipocytes and showed it is highly specific for GAS5 with no significant effect on other lncRNAs^27^. Briefly, GAS5 has a premature termination codon (PTC; region common to all GAS5 transcripts) which marks it for degradation by UPF1 via nonsense mediated decay^33^. We demonstrated that NPC86 disrupts UPF1 binding to GAS5 PTC and stabilizes GAS5 levels^27^. Here, we first characterized the binding of GAS5 to NPC86 using molecular dynamics (MD) simulation. Docking of NPC86 to the last 111 bp of GAS5 was performed as described in methods. The interaction of NPC86 with GAS5 was analyzed and root mean square deviation (RMSD) (Fig. 3a) and molecular docking glide score (Gscore) (Fig. 3b) results demonstrate that binding of NPC86 is robust and maintains conformational flexibility (Fig. 3c). Next, we evaluated the effect on GAS5 levels with NPC86 treatment in HT22 cells. We previously^27^ demonstrated that 20 nM was the optimal in vitro dose. Real time qPCR results (Fig. 3d) demonstrate that treatment with 20 nM NPC86 increased levels of GAS5 and insulin receptor in neuronal cells.

### Efficacy and safety of NPC86 in vivo in mice

**Figure 4:**
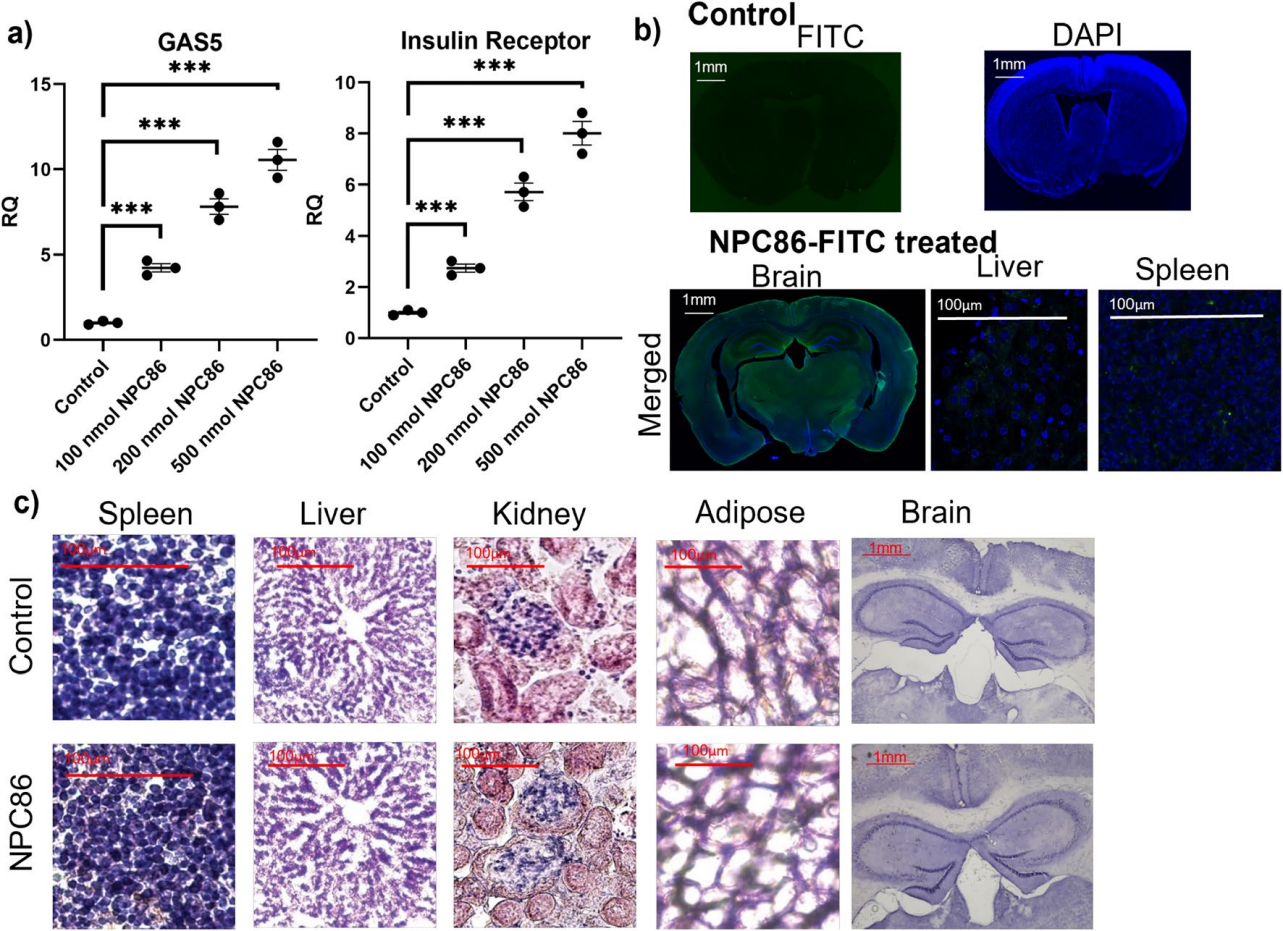
Efficacy and Safety of NPC86 in vivo in mice. (**a**) Increasing doses of NPC86 (100 nmol, 200 nmol or 500 nmol) or 100 nmol PBS vehicle (Control) was administered intranasally to young mice. RNA was isolated from hippocampus and qPCR performed using primers specific to GAS5 and insulin receptor and normalized to β-actin levels. Relative quantification (RQ) was determined using control as reference. Statistical analysis was performed by one-way ANOVA, ***p < 0.001. (**b**) All sections were treated with Sudan black B (SBB) dye to eliminate autofluorescence and nuclear counterstained with DAPI. Histochemistry of brain (scale bar 1 mm) from control mice and NPC86-FITC treated mice, liver (scale bar 100 μm), and spleen (scale bar 100 μm) from mice treated with NPC86-FITC (n = 5). c) Hematoxylin and eosin (H&E) staining of spleen, liver, kidney, adipose tissue (all with scale bar 100 μm) and brain (scale bar 1 mm) of control and 100 nmol NPC86-FITC treated mice (n = 5).

Based on the in vitro results, we next evaluated the effects of NPC86 in vivo in mice. The in vivo studies were initiated in young (6 months) C57BL/6J mice to evaluate the optimum dose of NPC86 as well as its toxicity and safety. To evaluate uptake and distribution, NPC86 was conjugated with FITC (NPC86-FITC) to enable visualization and we previously showed that NPC86-FITC functioned similarly as NPC86^27^. NPC86-FITC was delivered via the intranasal route to mice at 100 nmol, 200 nmol and 500 nmol concentrations. After 24 h, the hippocampus was analyzed using real-time qPCR and results (Fig. 4a) show a dose responsive increase in GAS5 and IR levels. Results indicated that 100 nmol was an optimal dose as our goal is to increase GAS5 to physiological levels and not have sustained over-expression which could be detrimental, as observed in certain cancers^34,35^. Coronal section of the brain from the 100 nmol NPC86 treated mice were imaged using Keyence BX810 microscope. Results (Fig. 4b) show that intranasal delivery of NPC86 crossed the blood brain barrier with distribution across all regions of the brain. Less than 5% of fluorescein label was detected in liver and spleen sections while it was not observed in the other organs. In addition, hematoxylin and eosin staining of brain (hippocampus), liver, spleen, kidney and adipose tissue sections indicated no toxicity in NPC86 treated mice (Fig. 4c).

### Administration of NPC86 to aged mice improves neuronal insulin signaling and decreases neuroinflammation in vivo

**Figure 5:**
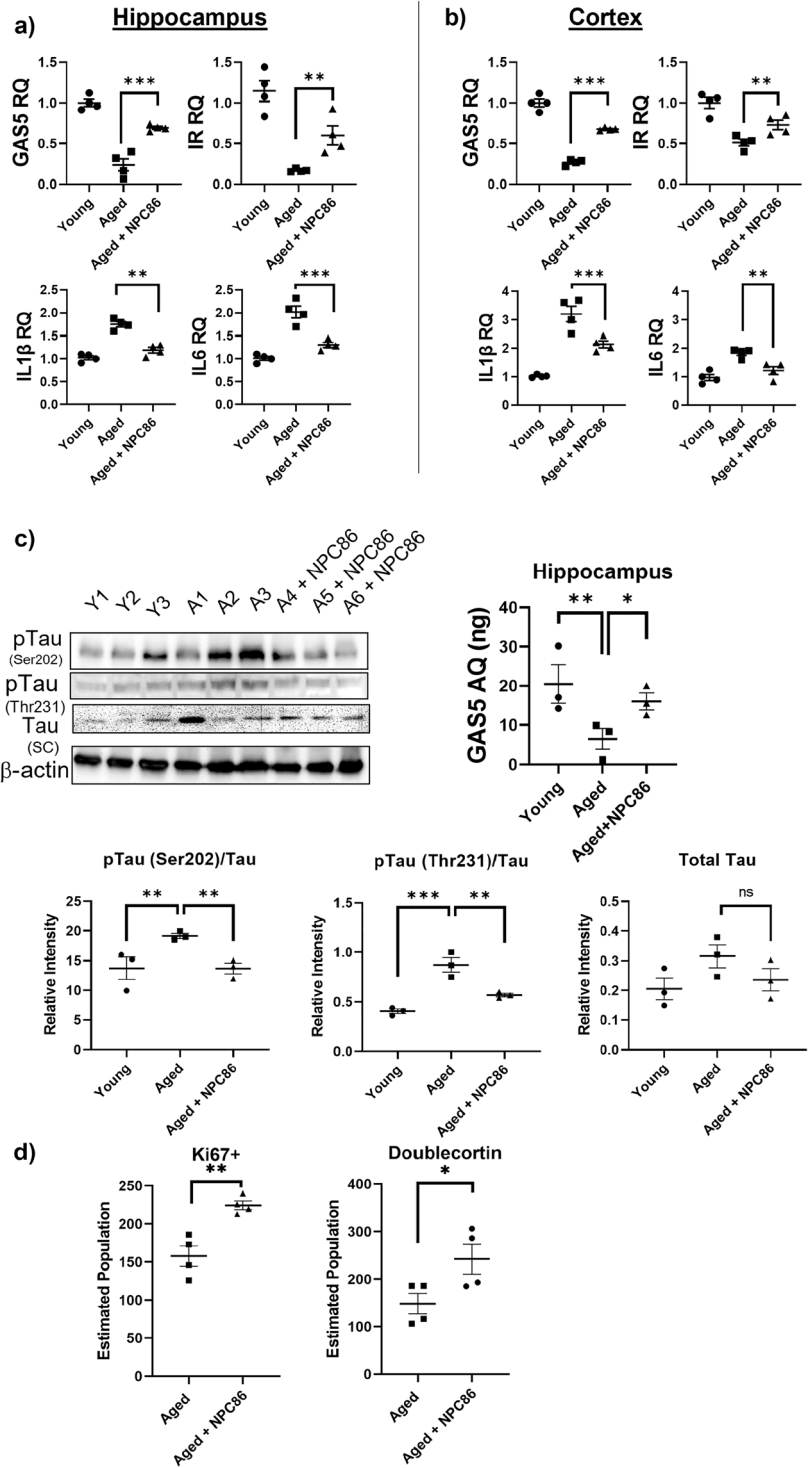
Administration of NPC86 to aged mice improves insulin signaling and decreases inflammation in vivo. 100 nM NP-C86 was administered intranasally to aged (20 months) C57BL/6J mice on alternate days (total 5 treatments). RNA was isolated on day 12 from (**a**) hippocampus and (**b**) cortex followed by qPCR analysis using primers for GAS5, insulin receptor, IL1β and IL6 and normalized to β-actin levels (n = 4). Relative quantification (RQ) was determined using young mice as reference. Each sample was run in triplicate. Statistical analysis was performed by one-way ANOVA, **p < 0.01, ***p < 0.001 comparing aged vs aged + NPC86. c) Hippocampal tissue was homogenized, and western blot analysis was performed on lysates using antibodies for pTau and total Tau as indicated (n = 3). Absolute quantification (AQ, ng) for GAS5 levels in hippocampal tissue of these mice. Densitometric analysis on the western blots was performed and pTau levels were normalized to total Tau. Statistical analysis was performed by one-way ANOVA, **p < 0.01, ***p < 0.001. d) The subgranular zone of the dentate gyrus was stained for Ki67 and doublecortin. Estimated population of Ki67 + and doublecortin in aged mice compared to aged + NPC86 was determined using the Stereo Investigator software (MicroBrightField, n = 4). Statistical analysis was performed by one-way ANOVA, **p < 0.01, *p < 0.05.

Since our results above (Fig. 1b) demonstrated that GAS5 levels are low in aged mice, we evaluated whether the small molecule therapeutic NPC86 could increase GAS5 levels and lead to improved neuronal insulin signaling and modulate neuroinflammation in aged mice. C57BL/6J mice (20 months) were administered 100 nmol NPC86 (Aged + NPC86) or vehicle PBS (Aged) via intranasal route on alternate days for a total of 5 treatments and tissues were analyzed on day 12. Young (6 months) mice were untreated and used as reference. Each cohort had 10 mice, equal M/F. The hippocampus and cortex were analyzed using real time qPCR. Results show that treatment with NPC86 significantly increased GAS5 levels in both hippocampus (Fig. 5a) and cortex (Fig. 5b) of aged mice concurrent with increase in insulin receptor levels. The inflammatory interleukins IL1β and IL6 were increased in the aged samples and treatment with NPC86 significantly decreased IL1β and IL6 levels.

Western blot analysis was performed on the protein lysates and arbitrarily picked three mice each per group is shown in Fig. 5c. Results show that tau phosphorylation on Ser202 (AD associated tau phosphorylation) is increased in aged mice (these are wild-type mice with no tauopathies) and NPC86 treatment decreases tau phosphorylation. Tau phosphorylation on Thr231 was higher in aged mice (though to a lower extent compared to phosphorylation of S202) and NPC86 treatment reduced it. Absolute quantification was determined by qPCR for GAS5 levels in these samples and results demonstrate that aged mice had lower GAS5 compared to young and treatment with NPC86 increased GAS5 levels in aged samples. Importantly, NPC86 treatment in aged mice increased proliferating progenitors as determined by Ki67 positive neurons, and increased newborn neurons as determined by doublecortin staining (Fig. 5d).

### RNAseq analysis of NPC86 treated aged mice

**Figure 6:**
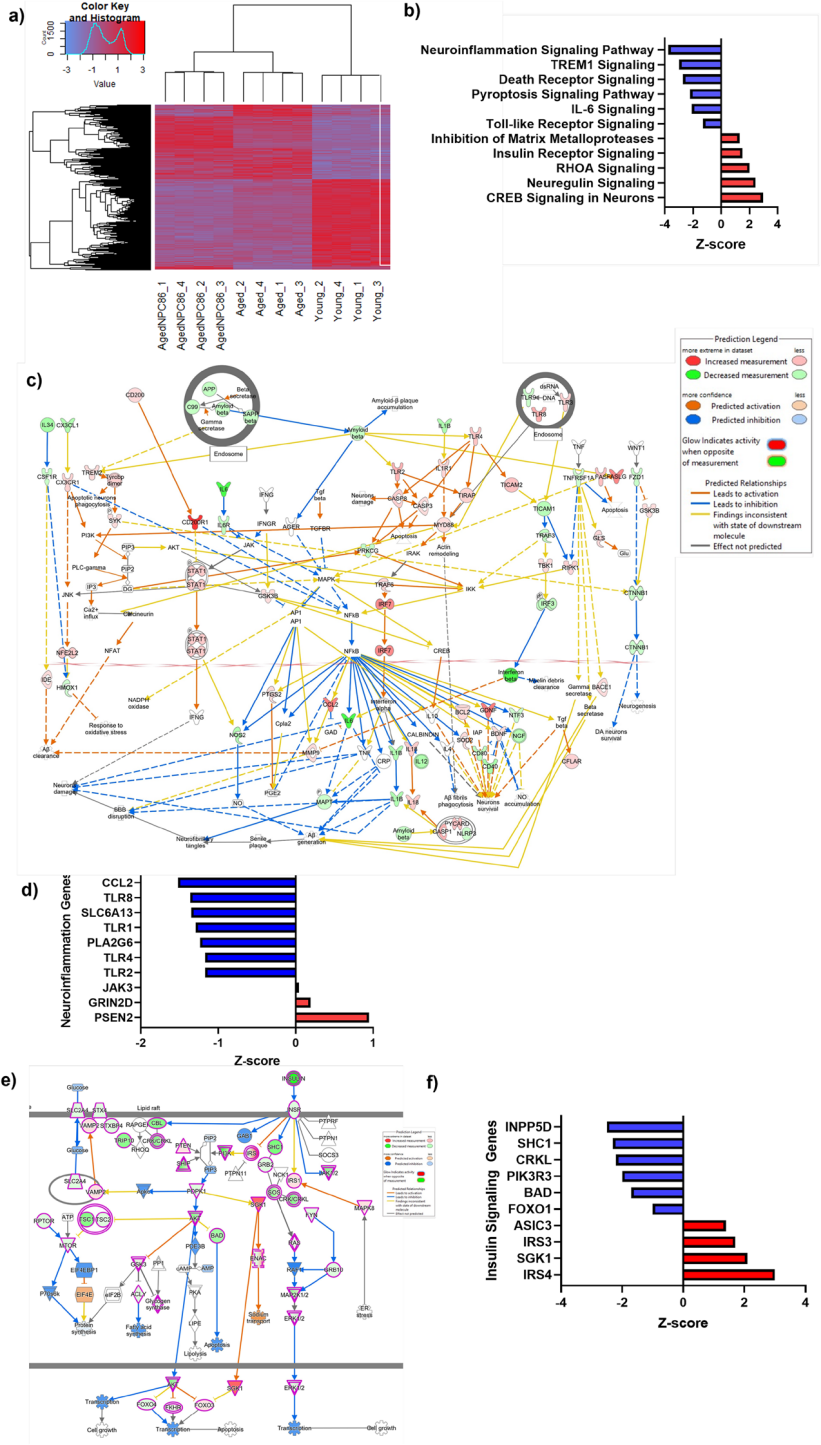

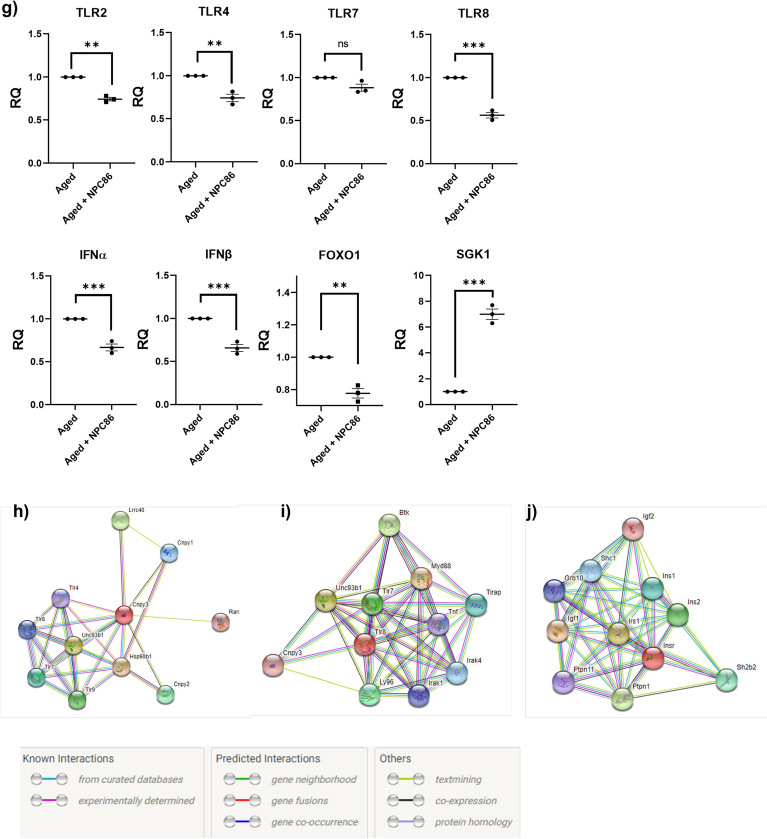
RNAseq Analysis of hippocampal tissue indicates NPC86 reduces neuroinflammation and increases insulin signaling in aged mice. (**a**) Heatmap shows hierarchical clustering of differentially expressed RNA transcripts in hippocampus of young, aged, and aged mice treated with NPC86 (2 FC, p-value < 0.05). Expression levels are represented by color and scale indicated in legend. (**b**) Graph of top ten canonical pathways significantly affected between aged and aged + NPC86 mice demonstrating the most upregulated (z-score > 1.3) and the most downregulated (z-score < −1.3) pathways identified by Ingenuity Pathway Analysis. Horizontal bars denote different pathways based on z-score. Red color indicates activation while blue color indicates suppression. (**c**) Neuroinflammation Pathway generated by IPA with changes in gene expression in a NPC86 dependent manner (**d**) top genes in neuroinflammation pathway downregulated (blue) and upregulated (red) identified by z-score between aged and aged + NPC86. (**e**) Insulin Signaling Pathway generated by IPA with changes in gene expression in a NPC86 dependent manner (**f**) top genes in IR pathway downregulated (blue) and upregulated (red) were identified by z-score between aged and aged + NPC86 mice. (**g**) QPCR of individual genes identified by RNAseq in the neuroinflammation and insulin signaling pathways, normalized to β-actin levels, with aged set as reference (n = 3). Statistical analysis was performed by one-way ANOVA, **p < 0.01, ***p < 0.001, and ns not significant. h) STRING analysis of protein associations with (**h**) TLR (**i**) TLR8 and (**j**) IR nodes.

Next, we sought to evaluate the transcriptomic effects of NPC86 treatment using mRNAseq analysis on hippocampal tissue from young, aged, and aged mice treated with NPC86. The RNAseq results (Supplemental data files) demonstrate that treatment with NPC86 increased GAS5 levels in aged mice while levels of other lncRNAs such as Malat1 and Neat1 were not affected. Heatmap with hierarchal clustering analysis (Fig. 6a) show differentially expressed mRNAs that changed significantly between young and aged, and we identified genes whose expression was reversed with NPC86 treatment compared to the age-related changes in Up, Down, Up (UDU) or Down, Up, Down (DUD) patterns. These were further identified and grouped into pathways using Ingenuity Pathway Analysis (IPA). Top ten canonical pathways that changed in response to NPC86 treatment in UDU or DUD pattern were identified that were distinguished by Z-score comparing aged mice to aged + NPC86 (Fig. 6b). The top pathway affected by NPC86 treatment was the Neuroinflammation Signaling Pathway (DUD pattern) and a network of the neuroinflammation pathway was created which included the genes that changed in a GAS5-dependent manner (Fig. 6c). Further analysis of the neuroinflammation pathways identified the top genes that were changed in aged mice and levels reversed with NPC86 treatment (Fig. 6d) in DUD and UDU patterns. The genes following the DUD pattern were CC motif chemokine ligand 2 (Ccl2), toll-like receptor 8 (Tlr8), solute carrier family 6 member 13 (Slca13: a neurotransmitter transporter), toll-like receptor 1 (Tlr1), gene encoding A2 phospholipase (Pla2g6), toll-like receptor 4 (Tlr4) and toll-like receptor 2 (Tlr2). The genes following the UDU pattern were presenilin 2 (Psen2), Glutamate Ionotropic Receptor NMDA.

Type Subunit 2D (Grin2d), and janus kinase 3 (Jak3); however, their z-score was less than 1 and p value was 0.05. Since our data demonstrated a significant increase in IR expression with NPC86 treatment (Fig. 5a,b above) and insulin signaling was identified in RNAseq analysis as one of the top signaling pathways activated by NPC86 treatment (UDU pattern), a network was generated comprising of top genes that changed downstream in the insulin signaling pathway (Fig. 6e,f). These genes, in the order of highest significance, and following the DUD pattern were inositol polyphosphate-5-phosphatase D (Inpp5d), Src Homology 2 Domain-Containing -SHC- Transforming Protein 1 (Shc1), Crk-like adapter protein (Crkl), Phosphoinositide-3-Kinase Regulatory Subunit 3 (Pik3r3), BCL2 Associated Agonist Of Cell Death (Bad), forkhead box protein O1 (Foxo1) and in the UDU pattern were insulin receptor substrate 4 (Irs4), serum-glucocorticoid regulated kinase 1 (Sgk1), insulin receptor substrate 3 (Irs3). These genes are downstream of the IR and are involved in crosstalk between signaling pathways.

The results of RNAseq were verified using real time qPCR (Fig. 6g) of representative genes in pooled RNA samples that were used in the RNAseq. Results demonstrate that NPC86 treatment in aged mice decreased the expression of Tlr2, -4, -7 and -8 with Tlr8 being the most significantly affected. The levels of Ifnα, Ifnβ, Foxo1 were concurrently reduced with NPC86 while Sgk1 expression increased with NPC86 treatment in aged mice. To understand the interactions of proteins in the IR and TLR pathways, STRING^36^ analysis was performed (Fig. 6h,i,j) and functional associations were visualized using IR, TLR and TLR8 as nodes.

### GAS5 regulates TLR8 expression

**Figure 7:**
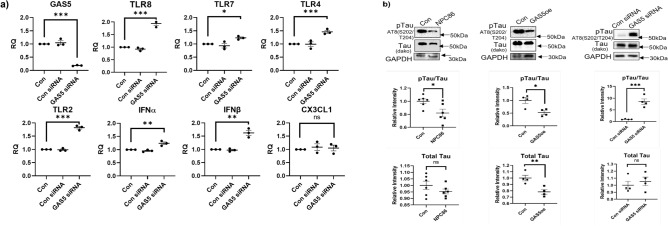
GAS5 regulates TLR8 expression and tau phosphorylation. (**a**) HT22 cells were transfected with GAS5 siRNA or scrambled control siRNA (Con siRNA) for 48 h. RNA was isolated, qPCR was performed, normalized to β-actin expression and relative quantification (RQ) was determined for GAS5, TLR8, TLR 7, TLR 4, TLR 2, IFNα, IFNβ, and CX3CL1 levels, with control set as reference (n = 3). Statistical analysis was performed by one-way ANOVA, *p < 0.05, **p < 0.01, ***p < 0.001. (**b**) HEK293T cells were transiently transfected to overexpress tauP301L. Cells were either treated with 20 nM NPC86 or co-transfected with GAS5 TOPO plasmid or transfected with 25 nM GAS5 siRNA. Western blot analysis was performed using antibodies for pTau and total Tau as indicated (n = 5). Densitometric analysis was performed, pTau and Tau levels were normalized to β-actin and pTau/tau relative intensity was calculated. Statistical analysis was performed by one-way ANOVA, *p < 0.05, **p < 0.01, ***p < 0.001, ns is not significant.

The RNAseq results indicated that toll-like receptor 2, -4, -7 and -8 levels were higher in aged mice and their levels decreased upon treatment with NPC86. To evaluate if GAS5 could directly affect the expression of TLRs, HT22 cells were depleted of GAS5 using siRNA. Real time qPCR results demonstrate (Fig. 7a) that depletion of GAS5 increased expression of Tlr8 by twofold while levels of Tlr2, -4, -7 were increased to a lower extent compared to Tlr8. Tlr8 modulates the expression of interferon α (Ifnα) and interferon β (Ifnβ). Hence, we evaluated the levels of Ifnα and Ifnβ in GAS5 depleted HT22 cells. Results show that Ifnα levels and Ifnβ increased in the GAS5 depleted cells. Cx3cl1 (fractalkine; modulated by Tlr2, -4) levels did not change significantly with GAS5 depletion.

### GAS5 regulates phosphorylation of human tau in vitro

The results of NPC86 administration in aged mice indicated a decrease in phosphorylation of tau. Tauopathy and hyperphosphorylation of tau are hallmarks of Alzheimer’s disease (AD) and other dementias such as FTDP-17. An FTDP-17-related mutant of human tau containing a proline to leucine mutation at position 301 (tau P301L) has been extensively studied as a model of tauopathy and is characterized by a faster phosphorylation rate than wild-type tau^37,38^. To evaluate an independent, direct effect of GAS5 on the phosphorylation of human tau, tau P301L plasmid was utilized. HEK293T cells were co-transfected with tau P301L plasmid along with GAS5-TOPO plasmid. Results (Fig. 7b) show significantly reduced phosphorylation of tau in the presence of GAS5, as detected by the AT8 antibody, an AD-associated tau epitope. Separately, GAS5 was depleted using GAS5 siRNA twenty-four hours prior to transient transfection of tau P301L plasmid, which revealed a dramatic increase in tau phosphorylation compared to control. Finally, NPC86 was added 24 h prior to transfection of tau P301L plasmid and maintained in medium. Results show decrease in phosphorylation of tau with NPC86 treatment.

### LPS chronic treatment decreases GAS5 levels in HT22 cells

**Figure 8:**
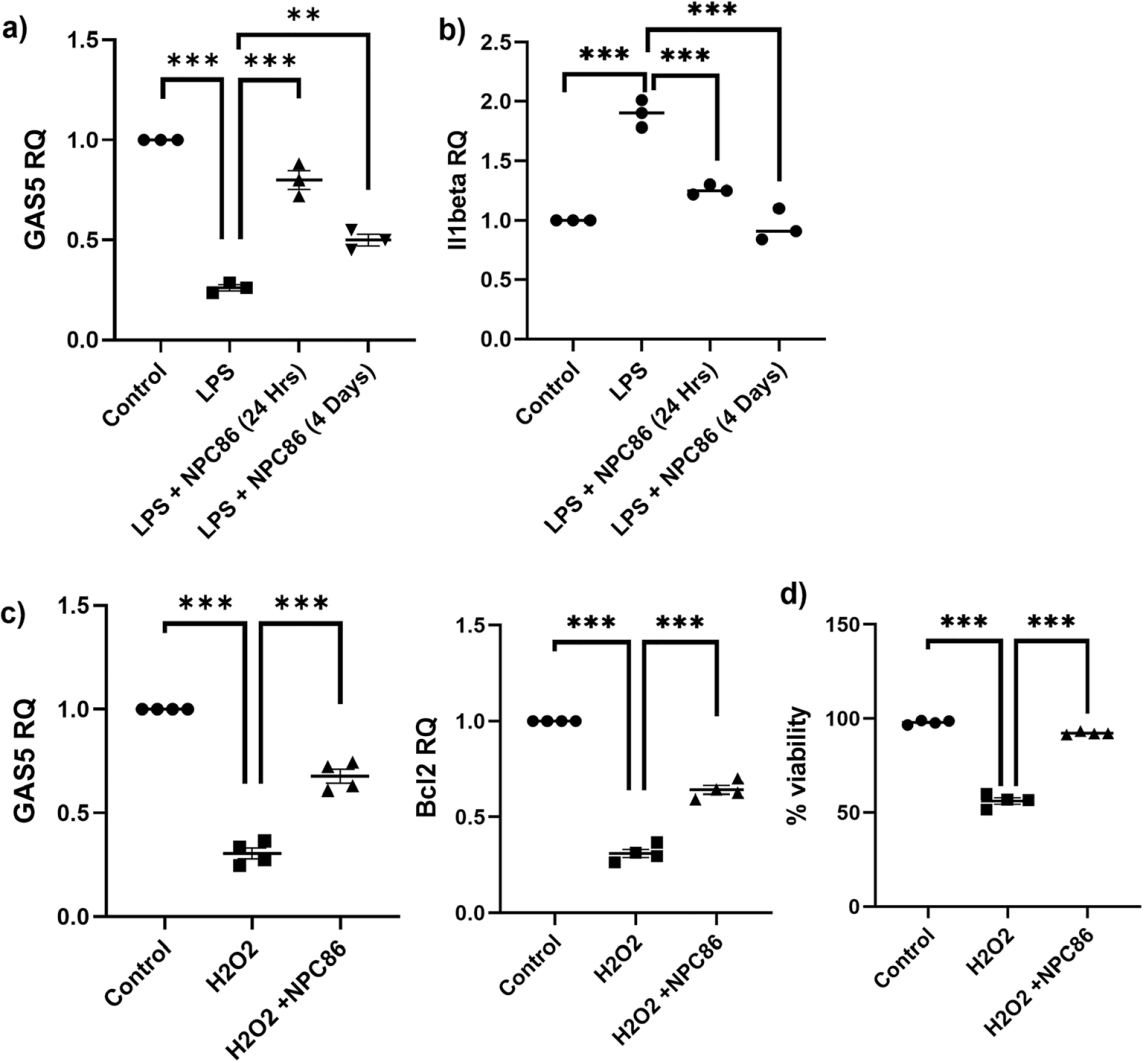
LPS chronic treatment decreases GAS5 in HT22. (**a**) HT22 cells were treated with LPS (5 ng/mL) for 4 days (LPS), HT22 cells were treated with LPS for 3 days followed by rescue with NPC86 treatment for 24 h (LPS + NPC86 (24 Hrs)) or NPC86 was added along with LPS for 4 days (LPS + NPC86 (4 days)). All cells were harvested on the fourth day, RNA was isolated, and qPCR was performed, normalized to β-actin expression and relative quantification (RQ) was determined for GAS5 and (**b**) IL1β, with control set as reference (n = 3). Statistical analysis was performed by one-way ANOVA, **p < 0.01, ***p < 0.001. (**c**) HT22 cells were treated with 100 μM H2O2 (1 h) followed by NPC86 treatment (7 h). RNA was isolated, qPCR was performed, normalized to β-actin expression and relative quantification (RQ) was determined for GAS5 and Bcl2 with control set as reference (n = 4). Statistical analysis was performed by one-way ANOVA, ***p < 0.001. d) HT22 cells were treated with 100 μM H2O2 (1 h) followed by NPC86 treatment (7 h). Acridine orange (AO) and propidium iodide (PI) dual staining was used to determine the viability of HT22 cells (*n* = 4). Statistical analysis was performed by one-way ANOVA, ***p < 0.001.

Chronic low-grade inflammation in the brain is a hallmark of aging and neurodegeneration. To mimic a microenvironment of low-grade inflammation, HT22 neuronal cells were treated with low levels of lipopolysaccharide (LPS; 5 ng/mL) for 4 days. Real time qPCR results (Fig. 8a) demonstrate that chronic treatment of LPS decreases GAS5 levels with concurrent increase of the inflammatory cytokine IL1β (Fig. 8b). We sought to determine if NPC86 could protect the cells from the LPS mediated inflammation. NPC86 (20 nM) was added to the cells along with LPS and maintained for 4 days (LPS + NPC86 (4 days)). Results demonstrate that co-treatment of cells with NPC86 inhibited LPS-induced decline in GAS5 cells and decreased IL1β levels. Separately, we sought to determine if treatment with NPC86 could rescue LPS mediated inflammation. HT22 cells were treated with LPS for 3 days, media changed and then administered NPC86 (20 nM) and cells were harvested on day 4 (LPS + NPC86 (24 h)). Results demonstrate that LPS induced decline of GAS5 was significantly rescued by 24-h treatment with NPC86 along with decrease in IL1β levels.

### H2O2 induced oxidative stress decreases GAS5 levels in HT22 cells

Oxidative stress is a key hallmark of aging. To evaluate the effect of oxidative stress on GAS5 levels, 100 μM H_2_O_2_ was added to HT22 cells for 1 h followed by treatment with NPC86 for 7 h. Results (Fig. 8c) demonstrate that H_2_O_2_ significantly decreases GAS5 levels with concurrent decrease in pro-survival Bcl2 levels and treatment with NPC86 post H_2_O_2_ increases levels of GAS5 and Bcl2. Separately, we evaluated HT22 cell viability using the AOPI assay. 100 μM H_2_O_2_ was added to HT22 cells for 1 h followed by treatment with NPC86 for 7 h. Results (Fig. 8d) demonstrate that treatment with NPC86 rescued the viability of cells concurrent with increase in levels of GAS5 and Bcl2.

## Discussion

The long noncoding RNAs (lncRNAs) have emerged as master regulators of gene expression and have diverse mechanisms of action that regulate transcription, splicing, translation and epigenetics. LncRNAs are greater than 200 nucleotides in length and are expressed in all cells in the nucleus or cytoplasm dependent on the cell and function. LncRNAs have distinct structural and spatial features which allow binding to DNA, RNA or protein partners. The lncRNA growth-arrest specific transcript 5 (GAS5) is a 5’-terminal oligopyrimidine (5′TOP) class of genes shown to regulate cell proliferation, growth and survival^24,25^. GAS5 was first evaluated in cancer cells where survival of tumors was attributed to GAS5 levels^39–42^. GAS5 is now shown to be expressed in all cells with varying functions depending on the cell type. Here, we demonstrated that GAS5 is abundantly expressed in the mouse brain, levels are reduced in aged brain and additionally, young diabetic, obese mice have lower GAS5 levels in the brain compared to young normal mice. Importantly, we also demonstrate that GAS5 levels are lower in human AD brain compared to age matched controls. A previous human transcriptomic analysis and microarray data of six brain regions from AD patients also showed that GAS5 is downregulated in AD^43^. Using variance-component transcriptomic-wide association studies (VC-TWAS) on the individual level and summary level genome-wide association study (GWAS) data, Tang et al. showed that GAS5 is a significant gene associated with Alzheimer’s disease and other neurological diseases^44^. Mouse and human GAS5 sequences are not highly conserved; however, lack of conservation in lncRNAs does not imply lack of conserved function^45^. The transcripts share 70% homology in exonic sequences^24,25^ but importantly, have conserved secondary structures across species which are critical to their function.

The results presented here demonstrate that lncRNA GAS5 is a viable target in brain during aging. The in vivo data in aged mice demonstrates NPC86 is nontoxic, crosses the blood brain barrier, increases GAS5 levels along with increased neuronal insulin signaling and decreased neuro-inflammation. We did not observe a dramatic improvement in cognition and behavior tests (not shown); however to evaluate this we will have to perform long-term administration of NPC86 during aging. Our study also showed that neurogenesis is increased upon NPC86 treatment. The RNAseq analysis provided a wealth of information on the GAS5-regulated genes and effects of NPC86 in aged brain. Insulin resistance accompanies metabolic syndrome, obesity and type 2 diabetes. Reduced levels of insulin receptors in the brain are seen in the elderly and in patients with dementia and Alzheimer’s disease^46^. Our data showed that Insr levels were lower in the aged mouse brain compared to young. NPC86 treatment in vivo significantly increased the Insr levels in hippocampus and cortex of aged brain while Igfr1 levels were not affected. RNAseq data (supplemental file) also demonstrated that Upf1 levels did not change and further other Upf1 targets such as Bcar1 and Nat9 did not change with NPC86 treatment. This was expected since NPC86 is specific for GAS5 and it disrupts binding of UPF1 with GAS5. The aged mice were wild-type C57BL6 and did not have any tauopathies. The phosphorylation of tau was higher in aged mice compared to young mice and NPC86 treatment decreased phosphorylation of tau in aged mice. Our data using HEK293T cells with tau P301L validated that GAS5 levels directly affect the phosphorylation of tau. Separately, we show that depletion of GAS5 in HT22 decreased phosphorylation of AKT concurrent with increase in tau phosphorylation. The GSK3β kinase is implicated in phosphorylation of tau; however our data in cells depleted of GAS5 did not show changes in the phosphorylation state of GSK3α/β. This suggests that the kinase phosphorylating tau in a GAS5-dependent manner is not GSK3α/β. Others have shown that AKT and CDK5^47^ or MARK^48^ mediate tau phosphorylation. Future studies utilizing kinase assays, mutations of phospho sites on tau will be undertaken by us to identify the GAS5-dependent kinase phosphorylating tau in neurons. Impaired insulin signaling also decreases glucose intake in the brain thereby decreasing Tau O-GlcNAcylation which also promotes hyperphosphorylation of tau^49^. Additionally, since insulin is not efficiently taken up due to decreased IR expression, excess extracellular insulin inhibits insulin degrading enzyme thereby reducing degradation of insulin and Aβ peptide^50^. Our data in DIO mice which present with insulin resistance showed that GAS5 levels were reduced in the hippocampus. We previously showed that intranasal insulin improves cognition and neuronal survival^51^. Prior research demonstrates that impaired insulin signaling sets the stage for Alzheimer’s disease (AD) and related dementias (ADRD)^52–57^. These results support the role of GAS5 in maintaining neuronal health via insulin signaling during aging.

Neuroinflammation signaling pathway was identified in RNAseq analysis as a significant pathway regulated by GAS5. The role of toll-like receptor (Tlr) in the CNS is associated with infection, inflammation and neurodegeneration. Expression of Ccl2, a chemokine, is modulated by the TLR pathway and results show Ccl2, Tlr8, -1,-2,-4 were increased in aged mice and then decreased upon treatment with NPC86. The in vitro experiments using GAS5 siRNA in HT22 cells demonstrated that Tlr8 increased dramatically with depletion of GAS5. TLR8 is activated by single stranded RNA and DNA. Ma et al. demonstrated that Tlr8 is present in the neurons and axons and further demonstrated that TLR8 functions in the neurons as an inducer of apoptosis and suppressor of neurite outgrowth^58^ and their results show that in neurons, Tlr8 does not signal through the canonical TLR pathway^59^. We are initiating studies to decipher the mechanism by which GAS5 regulates expression of TLR8. Here, our studies demonstrate that Tlr8 expression is regulated by GAS5 in the mouse neurons.

Reports pertaining to GAS5 roles in brain have conflicting viewpoints. GAS5 levels are shown to be increased in rodents following cerebral infarction and GAS5 binds to miRNAs such as miR-365a-3p, miR-137 in vitro^60,61^. Some studies reported upregulation of GAS5 in aged mice^62,63^ or beneficial effects of GAS5 silencing^64,65^. This may be explained by presence of alternatively spliced variants of the GAS5 gene. The biogenesis of GAS5 is established and is complex^25^. GAS5 gene transcribes several 10 box C/D small nucleolar RNAs (snoRNAs) (from the intervening intronic sequences) as well as multiple splice variants due to alternative splicing of the exons 1–10 of GAS5. While several splice variants have been reported or predicted computationally (ENSEMBL), due to presence of STOP codon, none of the transcripts are transcribed into protein and degrade via the nonsense-mediated decay pathway when translation is initiated^66^. The predominantly expressed splice variant is the full-length GAS5 variant with exons 1 through 12 spliced in sequential order. The last exon, exon 12, is present in all splice variants of GAS5 and is highly conserved between mouse and human. While the functions of all variants are not yet elucidated, it is demonstrated that splice variants have opposing function in cancer with the full-length variant enhancing cell proliferation and the splice variant promoting apoptosis^67^. Prior publications reporting increased GAS5 levels in brain of aging mice used primers or siRNAs targeting exons 2 through 8, region reported to undergo several different patterns of alternative splicing specific to the cell type and disease state^67–70^. To explain discrepancies, other researchers performed PCR using primer combinations to this region and products were separated on a gel. Results reveal splice variants of Exon 7 (7a/7b), exon 5 (5a/5b) and intron retention between exon 3 and 4^67,68^. We amplified the hippocampal RNA using different primer combinations between exon 2 through 9 and separated them on agarose gel. We observed several products suggesting multiple splice variants of GAS5 in the mouse brain. Determining the expression of possible splice variants in human and mouse brain in specific brain regions is an ongoing project of our lab. All our qRT-PCR experiments use primers contained within exon 12 which aids in evaluating the total expression of GAS5 incorporating quantification of any possible splice variants and enables reproducibility of results. Further, the in vitro experiments used siRNA targeting GAS5 exon 12 enabling depletion of all GAS5. Importantly, NPC86 binds to exon 12 which is highly conserved between mouse and human. Chen et al^71^ analyzed PBMC from aged samples and reported that circulating GAS5 levels can be correlated to aging. We have shown that GAS5 is secreted in exosomes from cells, detected in serum and disease state can change the ratio of amount retained by cell versus secreted^72^.

Neuroinflammation promotes pathology which sets the stage for Alzheimer’s disease and other neurodegenerative diseases such as Parkinson’s disease (PD), Amyotrophic lateral sclerosis (ALS) and Multiple sclerosis (MS). Lipopolysaccharide (LPS) is used as an endotoxin to induce inflammation to determine the in vitro cellular and molecular effects as well as in vivo to examine the behavior and neuronal pathology. It has been shown that neuroinflammation induced by LPS promotes deposition of amyloid and results in reduced spatial memory in vivo in rats^73–76^. In our study, low grade chronic inflammation induced by LPS in vitro resulted in significantly low GAS5 levels which was rescued by treatment with NPC86. These results suggest that GAS5 levels may be lowered by prevailing neuroinflammation and further, low GAS5 levels results in increased inflammation thereby exacerbating the environment. The molecular mechanisms underlying the cause of low GAS5 levels in brain in aging is being evaluated by our lab.

On a broader level, the study elucidates the role and targets of the regulatory lncRNA GAS5 in vivo in the aging brain using an RNA-targeting small molecule NPC86, a frontier in lncRNA-targeting therapeutic. NPC86 treatment improves neuronal insulin pathways and reduces neuroinflammation which are early determinants of ADRD pathology. In conclusion, we have demonstrated that GAS5 is an important, viable target in neurodegenerative diseases and NPC86 has a tremendous therapeutic potential in the aging process to prevent the advent of neurodegenerative diseases.

## Methods

### Cell Culture

Studies were carried out using immortalized clonal mouse hippocampal cell line (HT22) obtained from Dr. D.R. Schubert (Salk Institute). HEK293T cells (ATCC #CRL-3216) and HT22 cells were cultured in DMEM (Gibco # 11,965–092), 10% FBS (Sigma #F4135), and 1 × penicillin–streptomycin (Sigma #P4333) at 37 °C and 5% CO_2_.

### Tissue Analysis

The James A. Haley Veteran’s Hospital and University of South Florida Institutional Animal Care and Use Committee (IACUC) approved all experimental procedures with animals consistent with the governing guidelines and recommendations of AWA and HREA. All experiments complied with the ARRIVE guidelines. All mice were raised and studied in pathogen-free environments housed in plastic, sawdust-covered cages with normal light–dark cycle and free access to chow and water. Young (6 months) and aged (20 months) male and female C57BL/6J mice were purchased from Jackson labs and fed regular diets (10 kcal % fat diet from Research Diets D12450Bi). The diet-induced obese (DIO) mice and matched lean (4 months) male mice were purchased from Jackson labs and DIO were continued on a high fat diet (60 kcal % fat diets (Research Diets D12492i). The DIO mice (weight 30 g ± 3 g; blood glucose 389 ± 75 mg/dL; blood insulin 15 ± 2 ng/ml) were determined to be diabetic using IPGTT tests (AUC of blood glucose levels 625 ± 50 mg.ml/dL). Aged mice were treated with NPC86 intranasally as 5 μl drops in alternating nostrils while each mouse was held in supine position with its neck in extension. Mice were euthanized under CO_2_ anesthesia and cervical dislocation was used as a secondary means of mortality. Tissue was homogenized using a bead homogenizer to yield RNA or protein lysate for studies or processed for immunohistochemistry. RNA from human medial temporal lobe from Alzheimer’s disease (AD) patients or age matched normal (no cancers, 70–85 years, male and female, n = 9) were obtained from Byrd Alzheimer’s Institute (Dr. Bickford and Dr. Gordon).

### GAS5 depletion by siRNA

Silencer RNA targeting exon 12 of GAS5 was purchased (ThermoFisher cat#4,390,816, Assay ID#n272340; validated for efficacy, non-toxicity, specificity to eliminate off-target effects). HT22 cells were transfected with 25 nM GAS5 siRNA or control siRNA (negative control; scrambled sequence with no mRNA target) using RNAiMax reagent (ThermoFisher) for 48 h.

### Transient transfection

HEK293T cells were transfected using 2.5 µL Lipofectamine 2000 with 0.5–1 µg human tau 4R0N P301L plasmid (pRK5 vector backbone) as per experiment. 0.5 µg of either GAS5-TOPO plasmid or empty TOPO vector was co-transfected. Separately, 25 nM GAS5 siRNA used for depletion in tau P301L over-expressing cells and Scrambled siRNA used as control siRNA. Forty-eight hours after transfection, cells were harvested for western blot.

### Western Blot

Cell lysates were harvested using lysis buffer (Cell Signaling 9803S) + 10% protease/phosphatase inhibitor (Pierce A32957, A32953). Lysates were kept on ice or stored in the freezer for an hour, then sonicated briefly. Cell lysate (50 μg) were separated on SDS-PAGE gel, transferred to nitrocellulose membranes, blocked with 5% nonfat dried milk in Tris-Buffered Saline with 0.05% Tween 20 (TBST). Membranes were probed with pAKT (Ser 473, Cell Signaling #4058), AKT (Cell Signaling #2962), pGSK3β (Tyr 216/279, ThermoFisher #44-604G), GSK3β (Cell Signaling #9315), pGSK3α/β (Abcam #75,745), GSK3α/β (Abcam #131,356), pTau (Ser202, Cell Signaling #39,357), Tau DACO (Agilent A002401-2), pTau (AT8, Invitrogen MN1020B), Total tau (SantaCruz sc-32274) and β-Actin (Sigma #A3854). Secondary HRP antibodies for rabbit (Biorad #5196–2504) and mouse (Biorad #0300-0108P) were used with chemiluminesence incubation (Pierce #32,109) for detection. Images were digitally captured using ProteinSimple FluorChem™ and densitometric analysis was performed using AlphaView Software.

Automated western blot analysis using Simple WES system (ProteinSimple, Santa Clara, CA, USA) was used. The amount of lysate to antibody was optimized as per manufacturer’s instructions. A concentration of 0.4 mg/mL was found optimal to be used on all antibodies. The samples were separated on 12–230 kDa Wes Separation Module capillary cartridges of Simple Protein Wes system. PI3K kinase antibody sampler kit (Cell Signaling #9655) was used, and each antibody was used at a dilution of 1:50. GAPDH (Cell Signaling #D16H11) was used as a loading control (1:250 dilution of antibody). Anti-rabbit detection module kits were specific for Wes (ProteinSimple) and include Luminol-S, Peroxide, Streptavidin-HRP and anti-rabbit secondary antibody. The proteins are separated by capillary technology and analyzed based on the chemiluminescence signal peaks generated, shown as digital images representing bands as observed in traditional western blot analysis. Using Compass software (ProteinSimple), the peak areas of were estimated and normalized against GAPDH.

### Cloning of GAS5-TOPO plasmid

Full length GAS5 cDNA (spliced exons 1 through 12) was amplified by PCR using primers: Forward 3′GTTTCGAGGTAGGAGTCGACT5′ and reverse 3′ GGATTGCAAAAATTTATTAAAATTG3′. The product with 3′A tails was separated on a 1% agarose gel and a single band of 656 bp was verified. The amplified cDNA product was cloned into the TOPO-TA vector (ThermoFisher K452001). The insert and orientation were validated by sequencing.

### Quantitative polymerase chain reaction

RNA was isolated using Trizol™ (ThermoFisher #15,596,026) and cDNA was synthesized using 1 µg RNA (260/230 > 1.8 and 260/290 > 1.8) and iScript™ (Bio-Rad #1,708,891). Target was amplified with Maxima SYBR green/Rox qPCR master mix (Thermo Scientific #K0222) and qPCR was performed on the ViiA 7 (ABI). GAS5 primers were developed to amplify exon 12 to measure total GAS5 levels. Primer concentrations were optimized for a single melt curve and consistent amplification. Plate set up included a standard series, no template control and no reverse transcriptase control and samples were run in triplicate. A standard curve was generated for GAS5 and IR and used to calculate absolute quantities (AQ) of target expression normalized to β-Actin expression. Relative quotient (RQ) was determined using the comparative method (∆∆CT). Primers sequences are shown in the Table 1 below.

**Table 1:** Forward (F) and reverse (R) primer sequences shown 5′ to 3′ for genes amplified in qPCR with species specificity.

Gene	Species	Forward and reverse primer sequences (5′–3′)
β-Actin	Human	F: CTCTTCCAGCCTTCCTTCCT R: AGCACTGTGTTGGCGTACAG
β-Actin	Mouse	F: TGTCCACCTTCCAGCAGATGT R: AGCTCAGTAACAGTCCGCCTAGA
GAS5 Exon 12	Human	F: CTTCTGGGCTCAAGTGATCCT R: TGTGCCATGAGACTCCATCAG
GAS5 Exon 12	Mouse	F: CTCCTGTGACAAGTGGAC R: AACACAATATATCTGACACCATC
Insulin receptor	Mouse	F: AGATGAGAGGTGCAGTGTGGCT R: GGTTCCTTTGGCTCTTGCCACA
IL6	Mouse	F: TCCGGAGAGGAGACTTCACA R: TGCAAGTGCATCATCGTTGT
IL1β	Mouse	F: CTCGTGGTGTCGGACCCATATGA R: TGAGGCCCAAGGCCACAGGT
TLR8	Mouse	F: AAGTGCTGGACCTGAGCCACAA R: CCTCTGTGAGGGTGTAAATGCC
TLR2	Mouse	F: ACAGCAAGGTCTTCCTGGTTCC R: GCTCCCTTACAGGCTGAGTTCT
TLR4	Mouse	F: AGCTTCTCCAATTTTTCAGAACTTC R: TGAGAGGTGGTGTAAGCCATGC
TLR7	Mouse	F: GTGATGCTGTGTGGTTTGTCTGG R: CCTTTGTGTGCTCCTGGACCTA
CX3CL1	Mouse	F: CAGTGGCTTTGCTCATCCGCTA R: AGCCTGGTGATCCAGATGCTTC
IFNα	Mouse	F: GGATGTGACCTTCCTCAGACTC R: ACCTTCTCCTGCGGGAATCCAA
IFNβ	Mouse	F: GCCTTTGCCATCCAAGAGATGC R: ACACTGTCTGCTGGTGGAGTTC
SGK1	Mouse	F: CTCATTCCAGACCGCTGACAAAC R: CCAAGGCACTGGCTATTTCAGC
FOXO1	Mouse	F: CTACGAGTGGATGGTGAAGAGC R: CCAGTTCCTTCATTCTGCACTCG
GILZ	Mouse	F: CTAGCTCCGCAGGTGCGCAC R: CGAGGCCAACAGGTGAGCGG
AChE	Mouse	F: TTCCTTCGTGCCTGTGGTAGAC R: CCGTAAACCAGAAAGTAGGAGCC

### Sudan black B

Mice were anesthetized and transcardially perfused with 0.1 M phosphate buffered saline followed by 4% paraformaldehyde (PFA) in PBS. Brains were collected and placed in 4% PFA solution for 24 h and then were changed to 30% sucrose until completely sunk. 40 µm sagittal sections were prepared using a cryostat and stored in cryoprotectant solution. Brain sections were immersed in 0.1% Sudan Black B (SBB) dye (Sigma 34,197–25-5) and 70% ethanol for 20 min at room temperature. Excess dye was removed by rinsing 3 times with PBS. Sections were then processed by IHC.

### Histochemistry

Every sixth section was selected for all IHC procedures spanning the entire hippocampal region. Free floating sections were blocked with 10% normal goat serum with 0.1% Triton X-100 diluted in PBS for one hour. Tissues were then incubated in primary antibodies to doublecortin (DCX, Santa Cruz SC-8066; 1:200) or Ki67 (Novocastra, NCL-Ki67p; 1:500) diluted in PBS containing 3% goat serum and 0.1% Triton X-100 (PBS-TS) oscillating overnight at 4 °C. Secondary biotinylated antibodies were diluted 1:200 and 1:1000 in PBS-TS for DCX and Ki67, respectively, and incubated for 1 h at room temperature. Avidin–biotin complex was used to amplify signal for 1 h (Vector Labs) followed by color development with diaminobenzidine (Sigma) for DCX and diaminobenzidine with metal enhancer for Ki67. Cells labeled positively for either DCX or Ki67 were identified in the subgranular zone of the dentate gyrus. Estimated population was determined using the optical fractionator method for unbiased stereology and Stereo Investigator software (MicroBrightField). The hippocampus was sampled with a grid size and counting frame of 125 × 125 for both DCX and Ki67 in order to count all positive cells within the area of interest. Anatomical structures of interest were outlined using a 10x/0.45 objective and cells were counted using a 40x/0.95 objective.

Separately, liver, kidney, spleen, adipose tissue and coronal slices from the brain of aged mice treated with FITC conjugated NPC86 were sliced (40 μm) and fixed with mounting media with DAPI. Slides were imaged using the Keyence BZx810 microscope using the FITC and DAPI filters.

### Hematoxylin and eosin staining

Mice were perfused and organs were fixed and sliced (40 μm) as described above. Slices were stained with hematoxylin and eosin (H and E), dehydrated, cover-slipped and mounted on slides. Slides were imaged in brightfield using the Keyence BZx810 microscope.

### GAS5 in situ hybridization

Coronal brain slices were stained using RNAscope GAS5 probe or the negative control RNAscope bacterial dihydrodipicolinate reductase (DapB) probe (ACD kit #561630) using Akoya Opal secondary fluorophores per manufacturer’s instructions. Briefly, slices were mounted onto slides and permeabilized using RNAscope Pretreatment kit. GAS5 or dapB probe was hybridized, signal was amplified using RNAscope AMP reagent followed by labeling with Opal fluorophores. Slides were imaged using the Keyence BZx810 microscope or on a Zeiss AxioScan.Z1 (ZEISS Microscopy) slide scanner using a Plan-Apochromat 20x/0.8 M27 objective using the FITC and DAPI filters.

### Molecular dynamics (MD) simulation of GAS5 with NPC86

CENTROIDFOLD was used to generate a secondary structure prediction template for 3D RNA models generated by RNAComposer, which was used to model the last 111 base pairs of GAS5. This model was then minimized using Schrodinger Wizard after running Epik on the predicted structure prior to minimization. Molecular dynamics simulations were done on the last 111 base pairs using NAMD 2.12^4^ using the CHARMM36m force field^5^. Prior to simulation, the system was prepared using the CHARMM-GUI solution builder, with a salt concentration of 150 mM NaCl. Simulation parameters included constant pressure of 1 atm via Langevin dynamics, as well as a constant temperature of 310 K using Langevin piston Nosé − Hoover methods^6,7^. Long-range electrostatic forces were evaluated using the particle mesh Ewald (PME) with a 1 Å grid spacing^8,9^. Van der Waals interactions were calculated using a 12 Å cutoff with a force-based switching scheme after 10 Å, as well as a 2-fs time step integration via the SETTLE algorithm^10^. Analysis was done using VMD 1.9.3^11^ where the system was equilibrated for 10 ns restraining the Cα atoms of the protein (1.0 kcal/mol/Å^2^) to allow for solvation. This was followed by two production runs of 10 and 15 ns for the predicted 3D RNA structure of the last 111 base pairs of GAS5. Schrödinger’s Maestro program (version 9.3.5) was used as the primary graphical user interface and Maestro version 10.2 (Schrödinger, LLC, New York, NY) was used for virtual screening, which was performed on NPC86 prepared with Schrödinger’s LigPrep program (Schrödinger, LLC, New York, NY). The virtual screening method was performed using Schrödinger’s GLIDE software^12^ using the SP setting for the predicted 3D RNA structure of the last 111 bps, where compounds were docked on grids generated with Glide at a box determined by specifying nucleotide residues corresponding to a predicted binding region of NPC86 to GAS5 at RNA sequence “UAAUAAA”.

### RNAseq and ingenuity path analysis

RNA was isolated from hippocampus of young (6 months), aged (20 months), and aged + NPC86 treated mice. Three mice from each group were pooled to maximize biological diversity and sent for mRNA Sequencing. RNA concentration was measured, and quality checked using the Qubit and Agilent Tape station to insure RIN > 8.0. Library was prepared using the TruSeq stranded mRNA Library Prep Kit according to manufacturer’s instructions (Illumina #20040532) and resultant DNA library concentration and quality was checked again using the Qubit and Agilent Tape. Each library was pooled at equimolar concentrations and diluted prior to loading onto the flow cell of the Illumina NextSeq 500 with 75 bp pair-end reads with indices. Real-time image analysis and base calling were performed on the instrument using the NextSeq System Suite. All Samples had minimum 40 million reads and sequences aligned to > 80% to reference genome. Reads were trimmed using Trimmomatic and quality checked using FASTQC. In practice FASTQ files for each sample were split into four FASTQs to accelerate processing. Reads were mapped using HISAT2 to mouse genome GRCm39 (file downloaded from NCBI). Files were converted using SAMtools and FeatureCounts were used to determine reads. Differentially expressed genes were determined using R package DESeq2 and results were further investigated using Qiagen’s Ingenuity Path Analysis.

### AOPI survival assay

HT22 cells were grown in a 12-well plate and at 90% confluency treated with 100 μM H_2_O_2_ for 1 h, media changed and followed by NPC86 treatment. Cells were then trypsinized and washed once with PBS. The cell pellet (containing one million cells) was resuspended in 500 μL PBS and fixed by slow, drop-wise addition of 4.5 mL ice-cold 70% ethanol while gently vortexing. Samples were incubated overnight at 4 °C to complete fixation and then stored at − 20 °C until stained. A fresh solution of propidium iodide (1 mg/mL, VitaStainTM CS1-0109) and RNase A (2 mg/mL, Thermo Fisher EN0531) was diluted in water. Fixed cells were centrifuged at 1000 rpm for 5 min. The cell pellet was washed twice with PBS and the pellet was resuspended in 50 μL PI/RNase A solution and incubated at room temperature for 5 min. One milliliter PBS was added, and samples were divided to create unstained negative control for analysis. Acridine orange (VitaStainTM CS2-0106) was added (1:1) to samples for staining and incubated at 37 °C for 30 min then analyzed on the Nexcelom K2 cellometer.

### Statistical analysis

Densitometric analysis of western blots were analyzed using AlphaView™ software from ProteinSimple™. Experiments were independently repeated three to five times for reproducibility. PRISM software (GraphPad) was used for statistical analysis. Two-way analysis of variance (ANOVA) or Student's t test was used in the analysis indicated by the figure legends (*P < 0.05, significant; **P < 0.01, highly significant; ***P < 0.001, extremely significant).

## Supplementary Information

Supplementary Information 1.

Supplementary Information 2.

Supplementary Information 3.

## Data Availability

The dataset generated and analyzed during this study is included in the published article and its supplementary information file.
