# Supplementary material for: Small molecule targeting long noncoding RNA GAS5 administered intranasally improves neuronal insulin signaling and decreases neuroinflammation in an aged mouse model
Source: Sci Rep. 2023 Jan 6;13:317. doi: 10.1038/s41598-022-27126-6 (PMC9822944; doi:10.1038/s41598-022-27126-6)
Supplement: Supplementary file 1 — Supplementary Information 1. [file 41598_2022_27126_MOESM1_ESM.docx]

**Supplemental data**

Small molecule targeting long noncoding RNA GAS5 administered intranasally improves neuronal insulin signaling and decreases neuroinflammation in an aged mouse model.

Rekha S. Patel^1^, Ashley Lui^2^, Charles Hudson^1^, Lauren Moss^3^, Robert P. Sparks^4^, Shannon E. Hill^2,6^, Yan Shi^5^, Jianfeng Cai^5^, Laura J. Blair^1,2,6^, Paula Bickford^1,3^, Niketa A. Patel^1,2,7^

Contents

Excel sheet: RNAseq FASTQ data of genes in samples of hippocampus from young, aged and aged+NPC86 mice.

RNAseq FPKM data and full-length western blots of panels shown in figures 2b, 5c and 7b.
