# Supplementary material for: Small molecule targeting long noncoding RNA GAS5 administered intranasally improves neuronal insulin signaling and decreases neuroinflammation in an aged mouse model
Source: Sci Rep. 2023 Jan 6;13:317. doi: 10.1038/s41598-022-27126-6 (PMC9822944; doi:10.1038/s41598-022-27126-6)

Original Western blots for panels shown in Figures 2b, 2c, 5c and 7b

Figure 2b pAKT

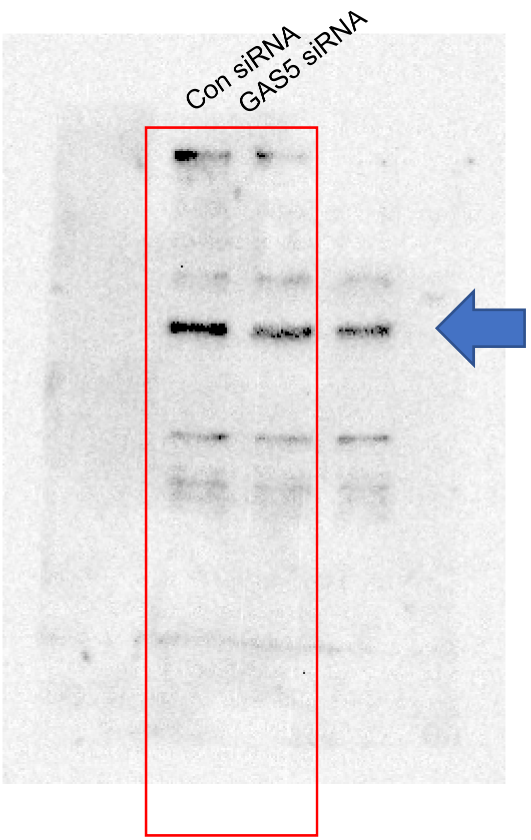

Figure 2b AKT

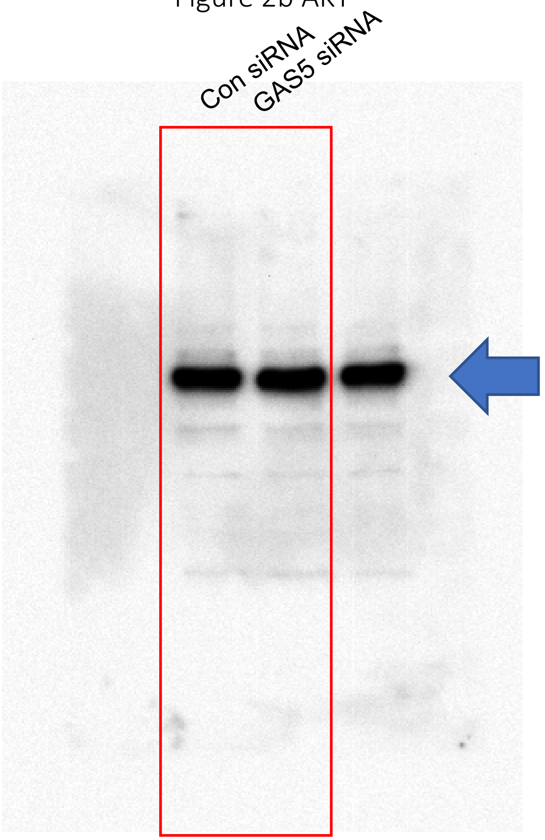

Actin

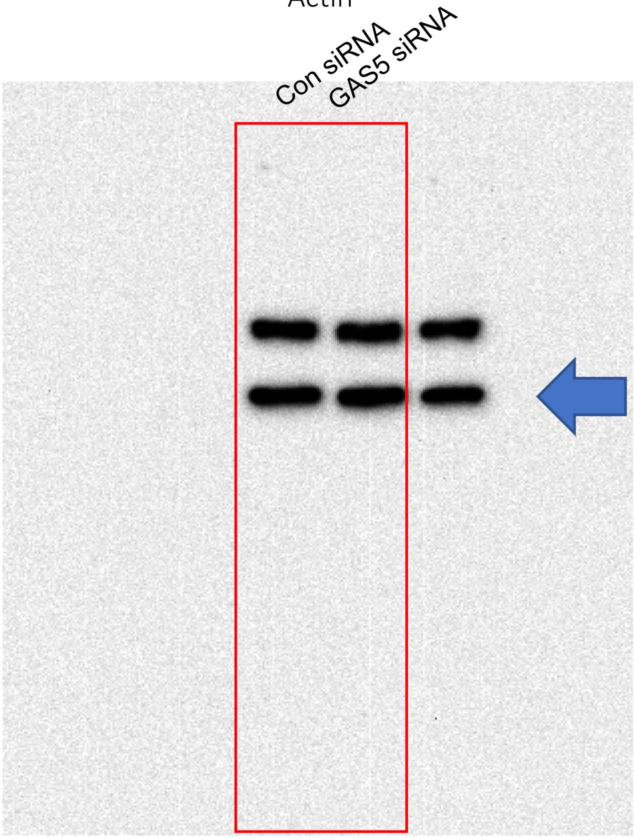

Figure 2b pTau Ser202

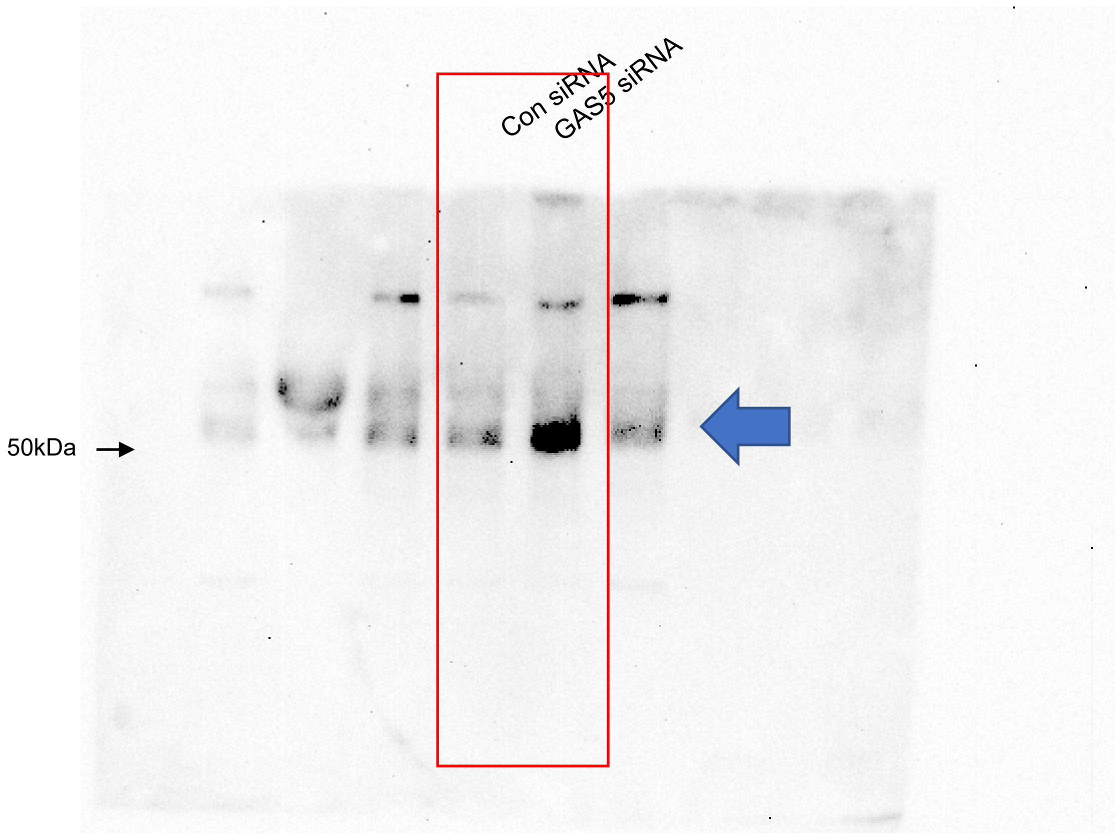

Figure 2b Tau

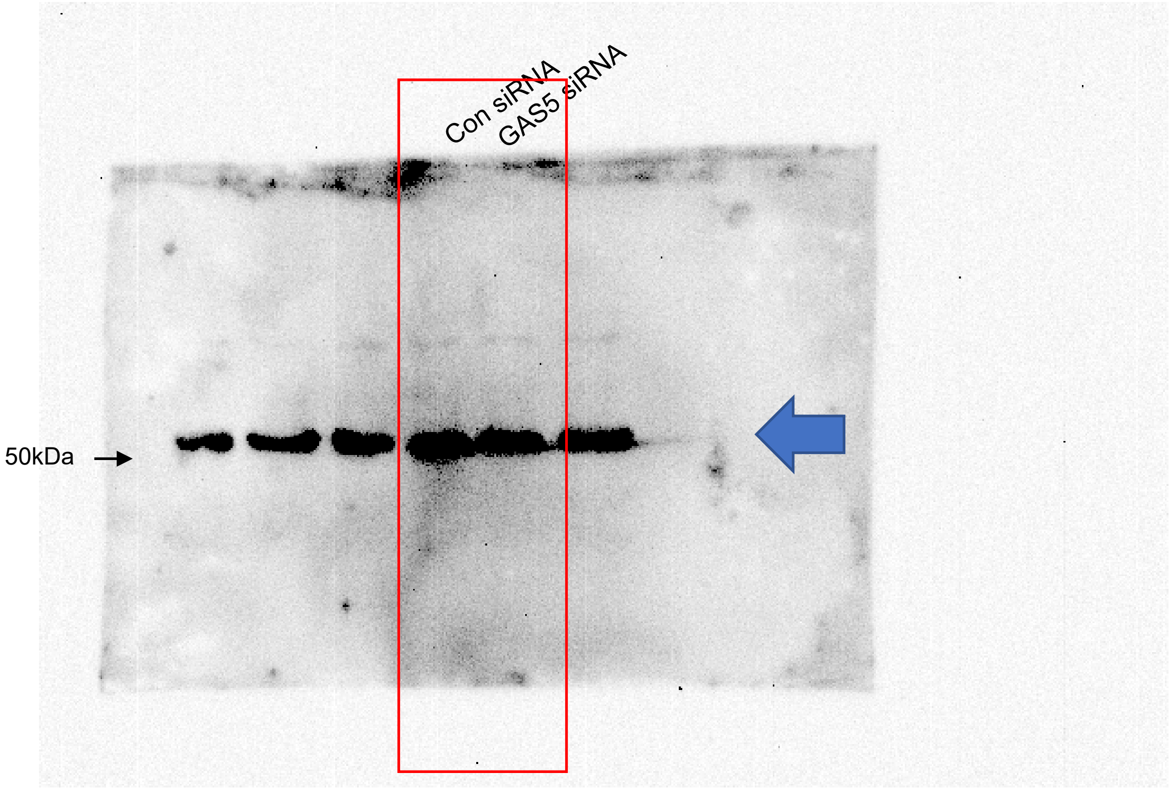

Figure 2b Insulin receptor (IR)

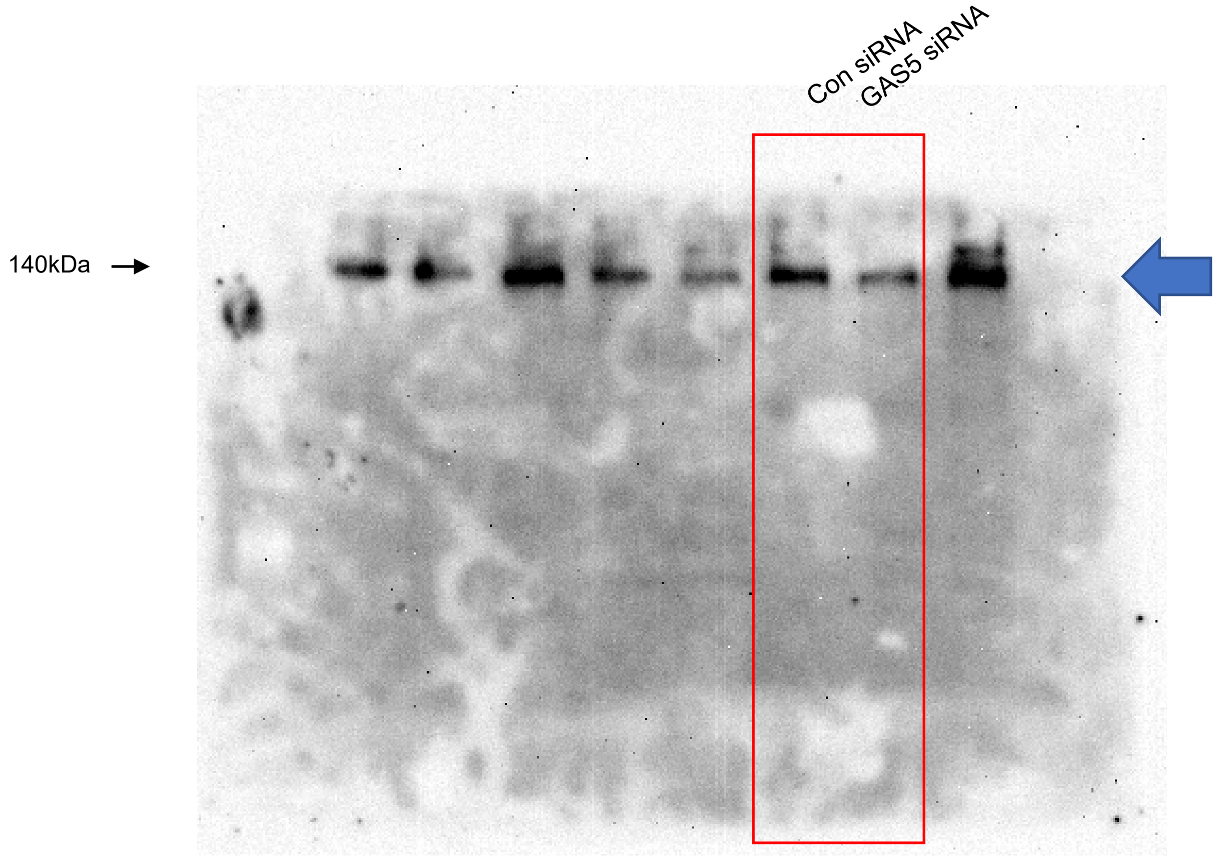

Figure 2b
pGSK3b Tyr216/279

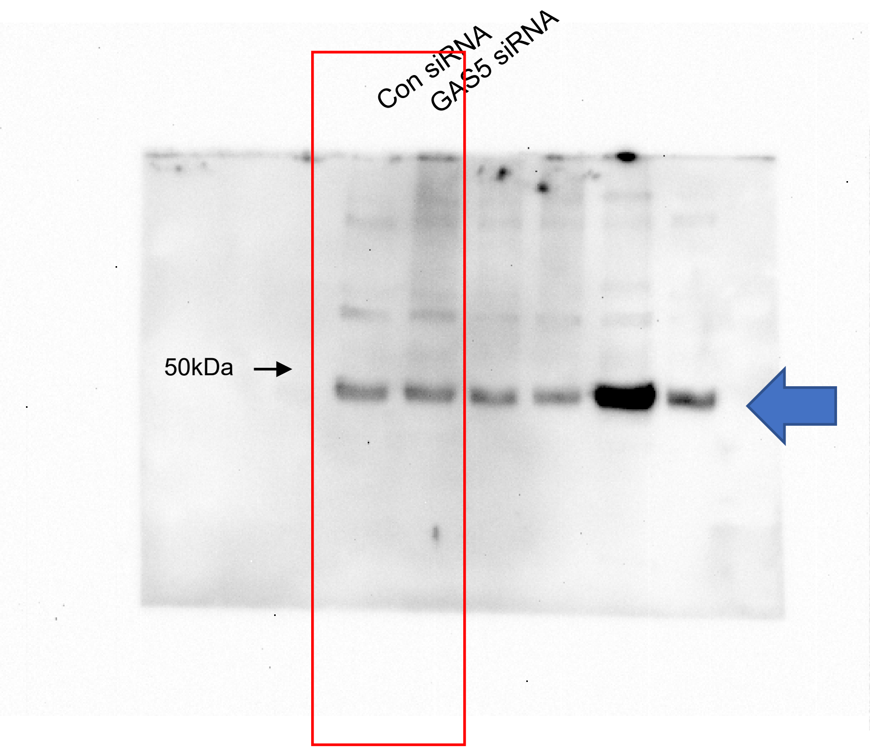

Figure 2b pGSK3a/b

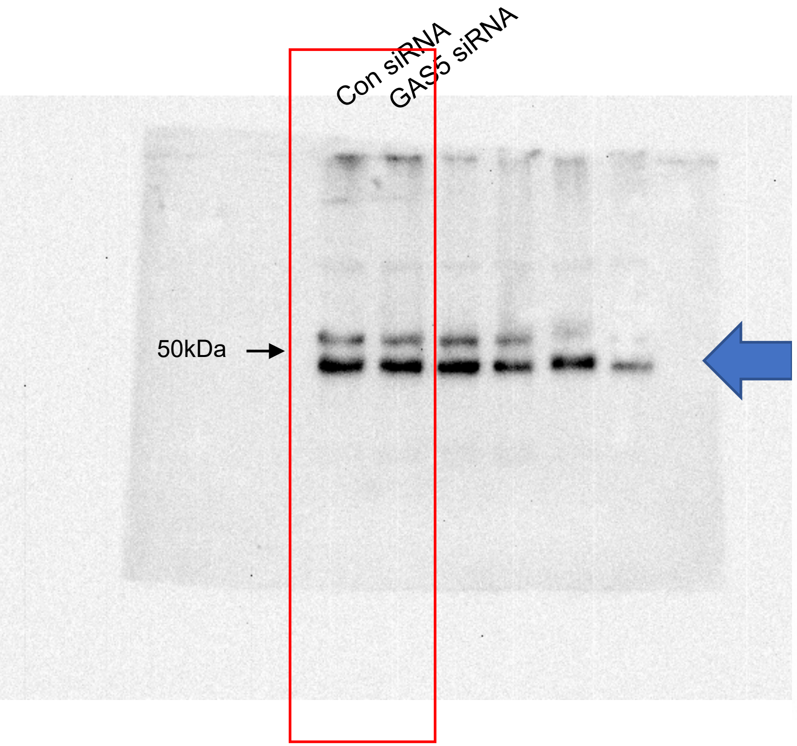

Figure 2b GSK3a/b

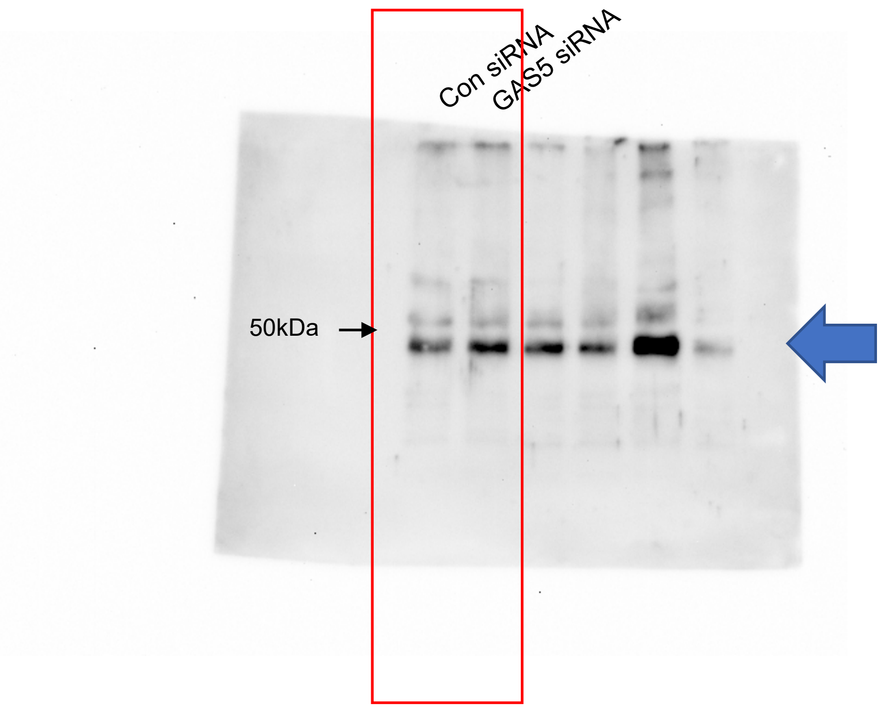

Figure 2b Actin

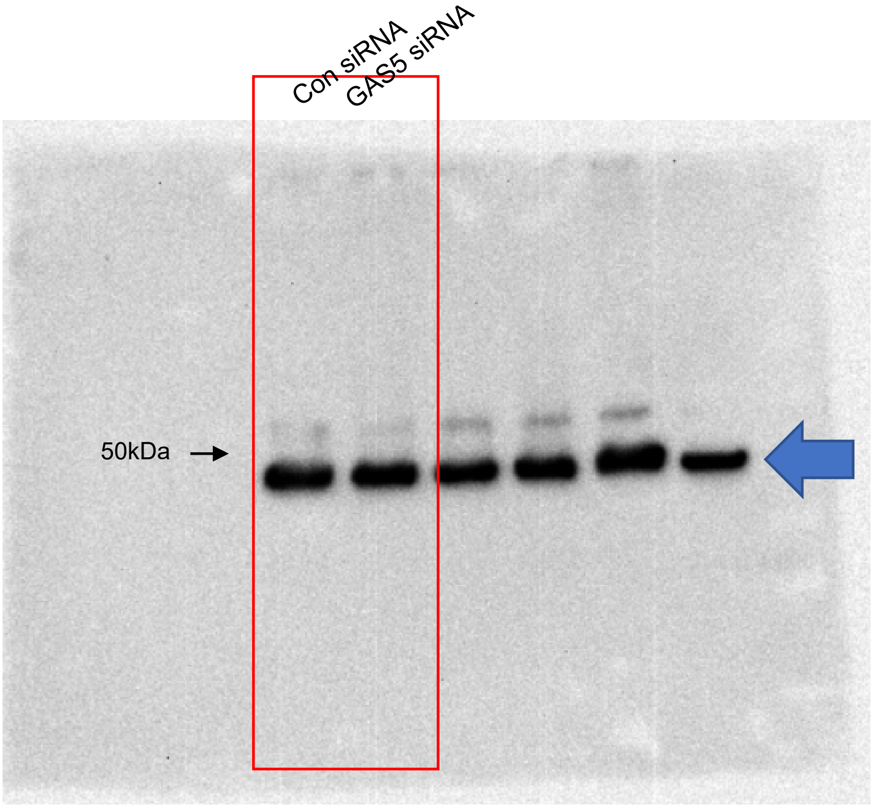

Figure 5c pTau Ser 202

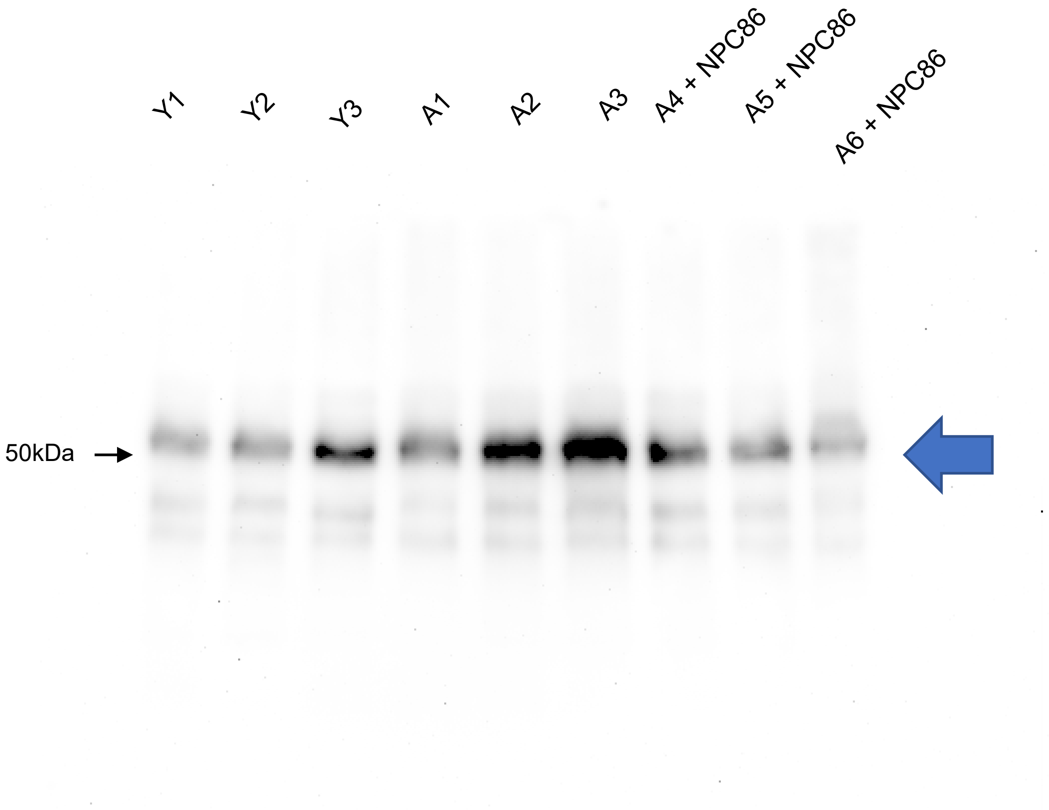

Figure 5c Tau santa cruz

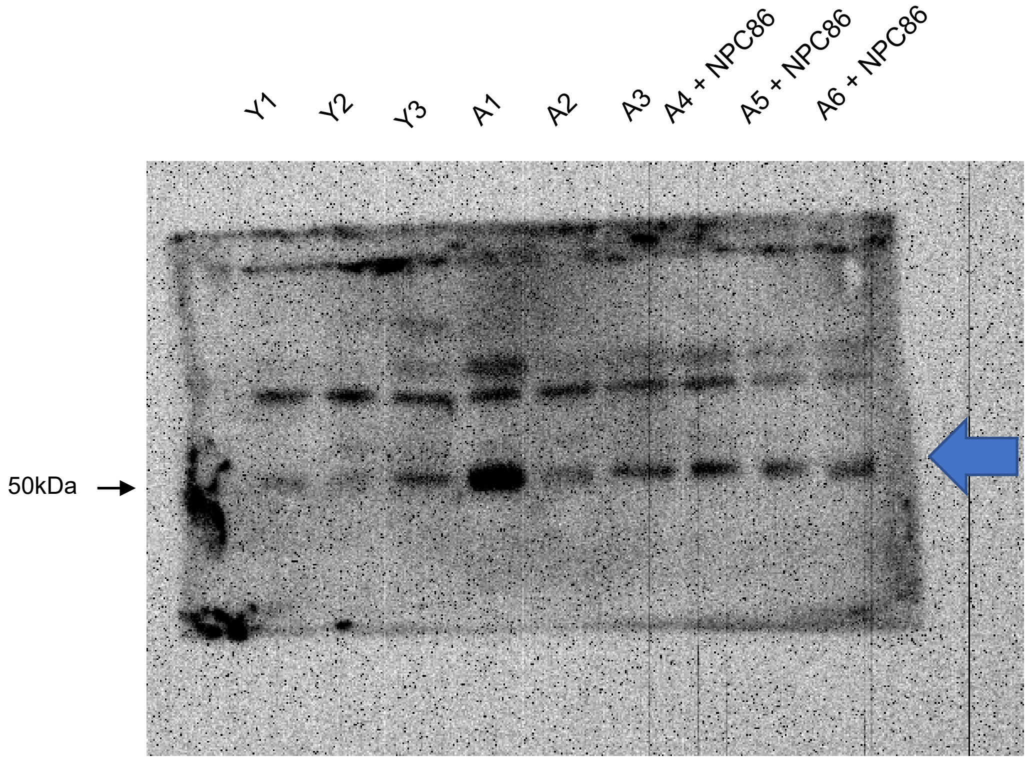

Figure 2c Automated western blot using WES (ProteinSimple)

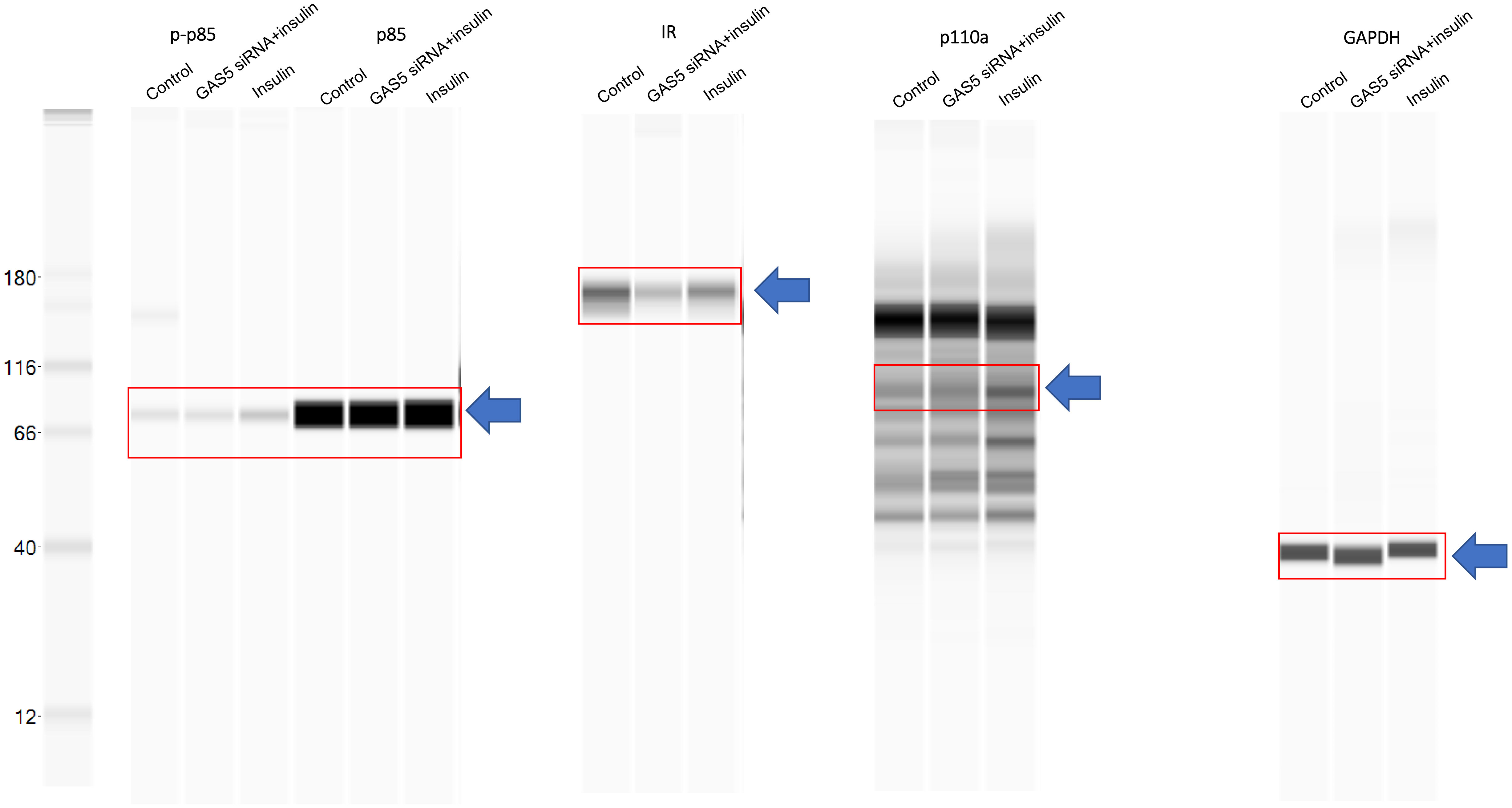

Figure 5c pTau Thr 231

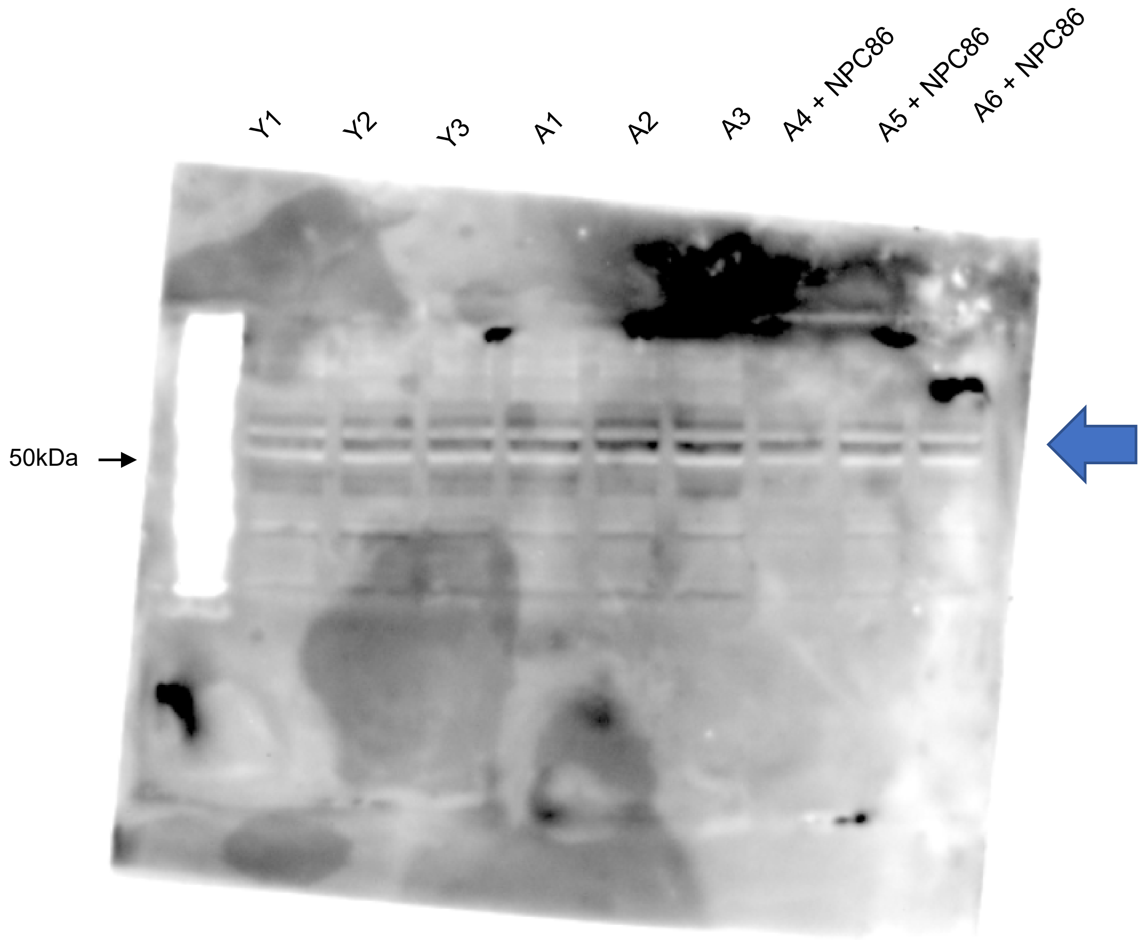

Figure 5c Actin

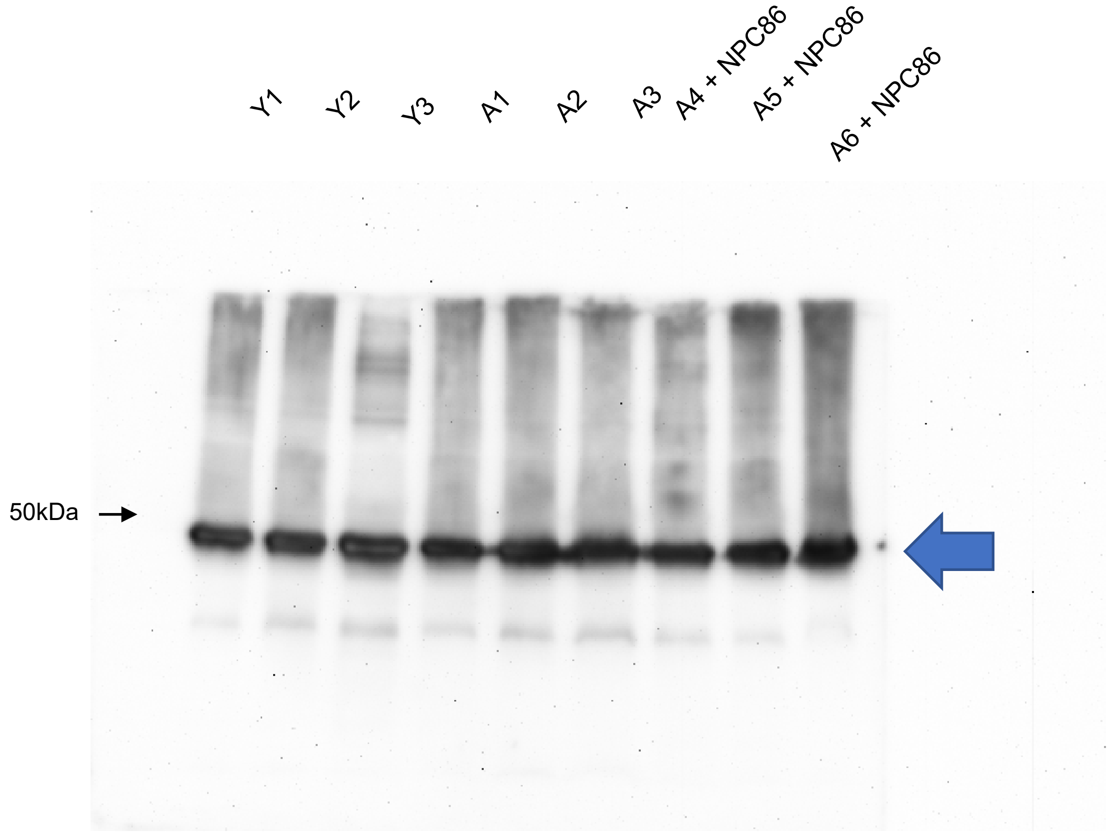

Figure 7b pTau AT8(Ser202/Thr204)

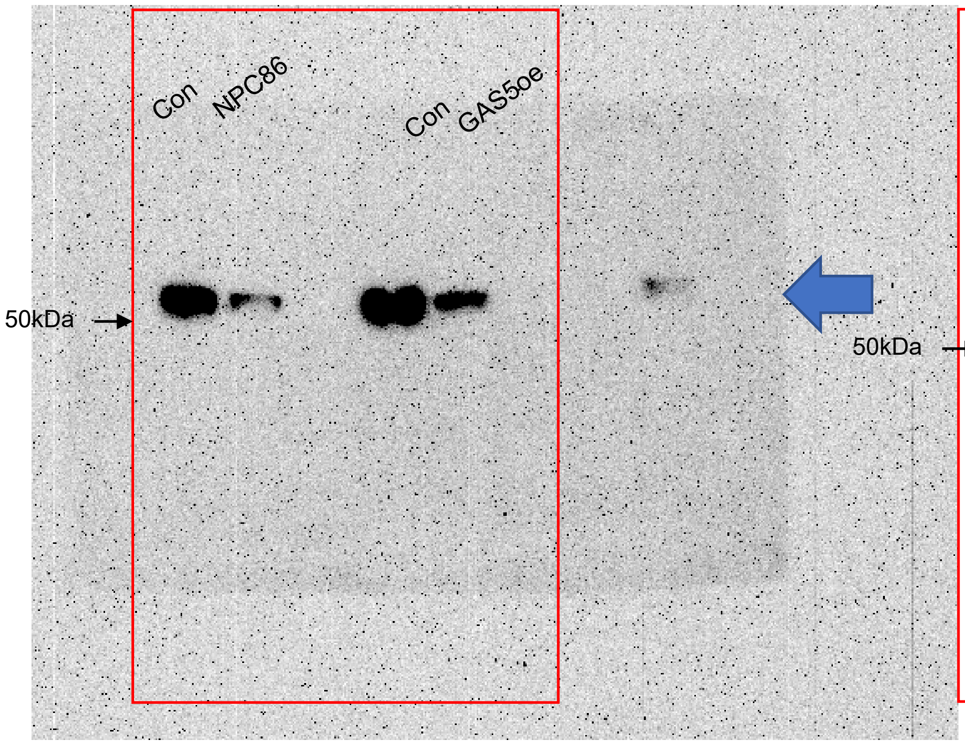

Figure 7b Tau Dako

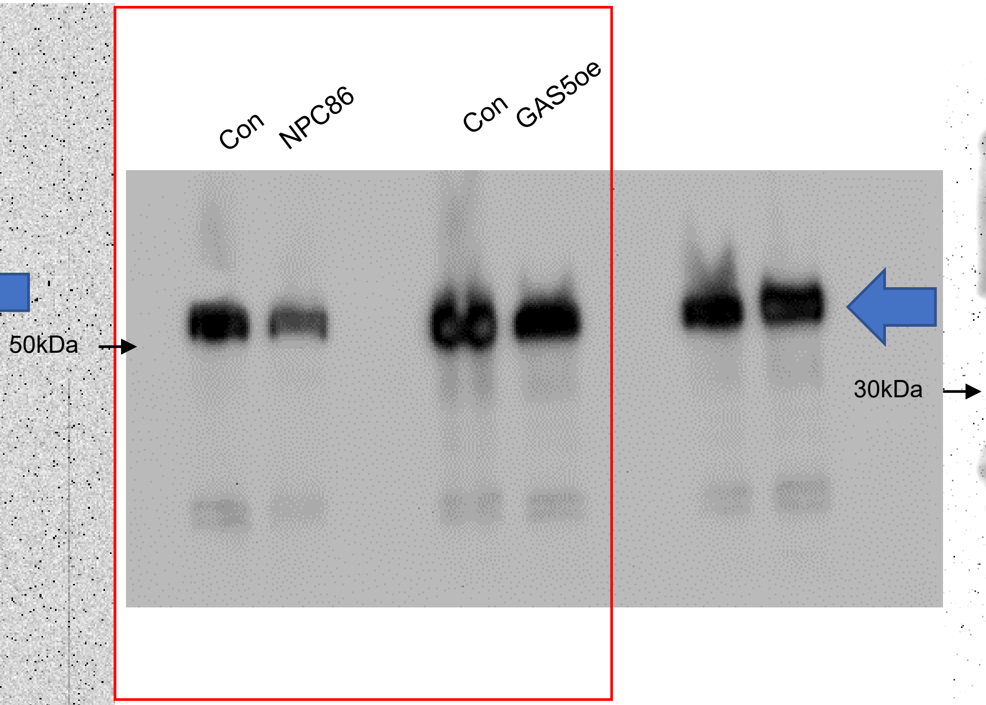

Figure 7b GAPDH

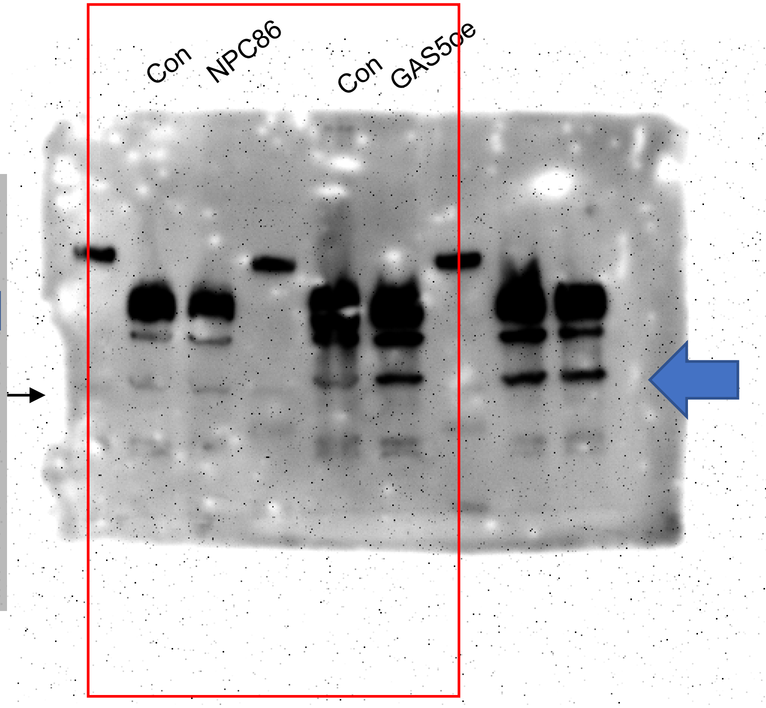

Figure 7b pTau AT8(Ser202/Thr204)

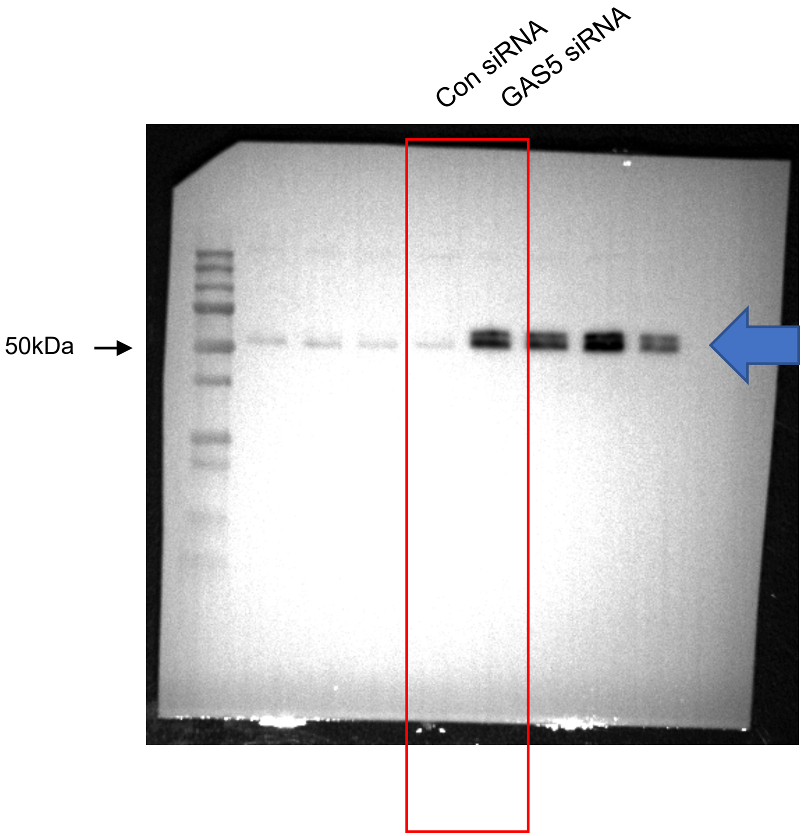

Figure 7b Tau Dako

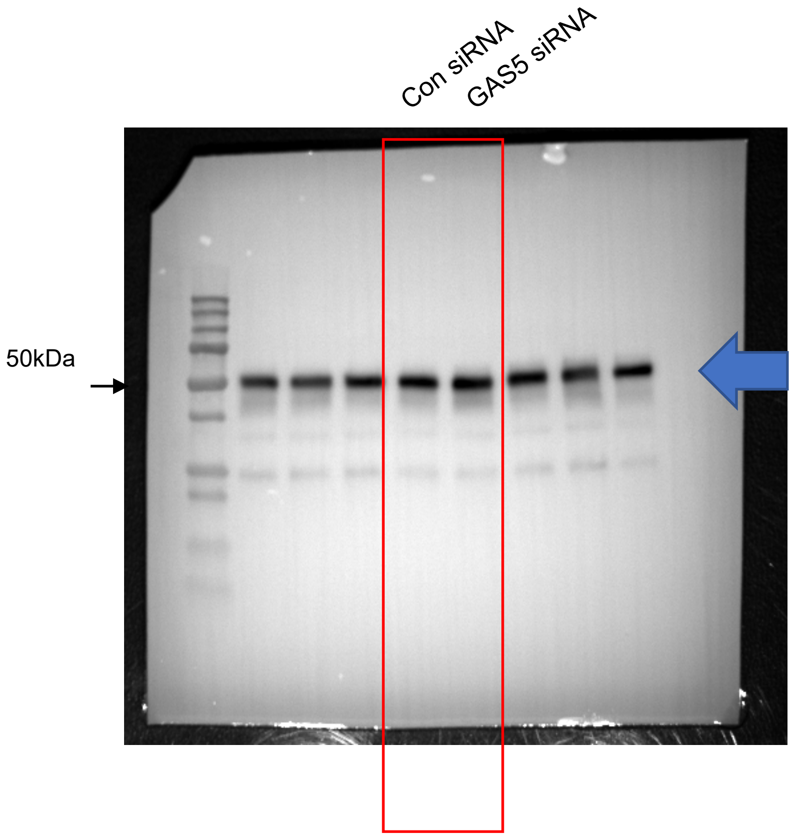

Figure 7b GAPDH

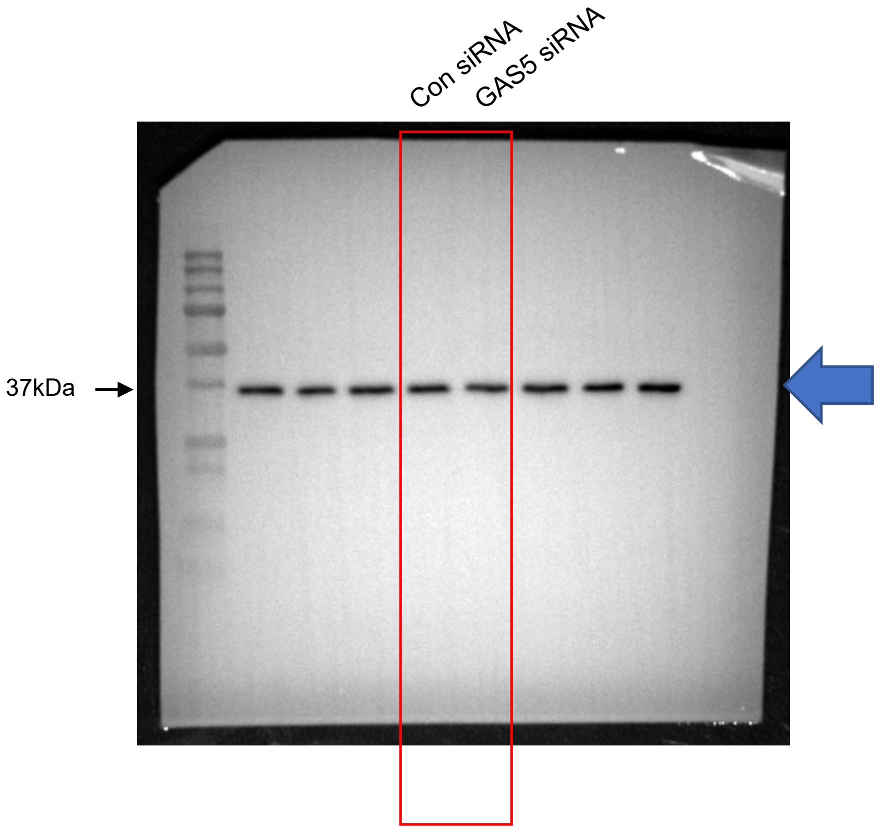

Supplement: Supplementary file 3 — Supplementary Information 3. [file 41598_2022_27126_MOESM3_ESM.pdf]
